# Supplementary material for: Prevalence and intensity of soil-transmitted helminth infections in Narok and Bomet Counties, Kenya: Evidence from program monitoring
Source: PLoS Negl Trop Dis. 2025 Dec 30;19(12):e0012415. doi: 10.1371/journal.pntd.0012415 (PMC12753061; doi:10.1371/journal.pntd.0012415)
Supplement: Attachment — Submitted filename: PNTD-D-24-01087_reviewer_GC_30.12.2024.docx [file pntd.0012415.s002.docx]

**PLOS Neglected Tropical Diseases**

**Prevalence and intensity of soil-transmitted helminth infections in Narok and Bomet Counties, Kenya: Evidence from program monitoring**

--Manuscript Draft--

| **Manuscript Number:** | PNTD-D-24-01087 |
| --- | --- |
| **Full Title:** | Prevalence and intensity of soil-transmitted helminth infections in Narok and Bomet Counties, Kenya: Evidence from program monitoring |
| **Short Title:** | Prevalence and intensity of soil-transmitted helminth infections in Kenya |
| **Article Type:** | Research Article |
| **Keywords:** | Helminthiasis, Public Health, Prevalence, Morbidity, kenya |
| **Abstract:** | Background  The backbone of Kenya's soil-transmitted helminthiasis control program is the periodic distribution of anti-helminthic Preventive Chemotherapy (PC). PC distribution has been conducted since 2012 through the National School-Based Deworming Programme, which deworms school-age children (SAC, 5–14 years) living in areas at high risk for soil-transmitted helminth (STH) infections. After nearly a decade of deworming, the Ministry of Health sought to generate evidence for program monitoring by estimating the prevalence and intensity of STH infection in Narok and Bomet counties among at- risk groups: preschool-aged children (PSAC, 2–4 years), SAC, and women of reproductive age (WRA, 15–49 years).  Methods  During August and December of 2021, we conducted population-based household surveys in Narok and Bomet counties, respectively, using a multi-stage, cluster random sample among resident PSAC, SAC, and WRA. Individual and household questionnaires were administered using an electronic mobile platform. Stool samples were collected and tested for roundworms (Ascaris lumbricoides), hookworms (Necator americanus, Ancylostoma duodenale), and whipworms (Trichuris trichura) using duplicate thick-smear Kato-Katz microscopy.  Results  Stool samples were provided by 1,062 PSAC, 1,922 SAC, and 364 WRA. Results indicated that the prevalence of any STH infection was highest among SAC (16.2% [upper 95% design-corrected confidence interval [U95% CI]: 23.1%]), followed by PSAC in Bomet county (15.8% [U95% CI: 21.7%]). In Narok county, STH prevalence was highest among PSAC (12.8% [U95% CI: 18.2%]) followed by SAC (11.3% [U95% CI: 16.2%]). Moderate-to-high intensity infection prevalence among PSAC and SAC exceeded the morbidity elimination threshold of 2% in both counties. A. lumbricoides and T. trichura were most identified; few hookworm infections were detected.  Conclusions  STH infections remain a public health problem in Narok and Bomet counties. There may be a need to expand PC distribution to include risk groups beyond SAC during deworming exercises, as envisioned in the Breaking Transmission Strategy, to hasten progress toward the achievement of STH as a public health problem. |
| **Additional Information:** |  |
| **Question** | **Response** |
| **Government Employee**  Are you or any of the contributing authors | No - No authors are employees of the U.S. government. |

| an employee of the United States government?  Manuscripts authored by one or more US Government employees are not copyrighted, but are licensed under a [CC0](https://creativecommons.org/about/cc0) [Public Domain Dedication](https://creativecommons.org/about/cc0), which allows unlimited distribution and reuse of the article for any lawful purpose. This is a legal requirement for US Government employees.  This will be typeset if the manuscript is accepted for publication. |  |
| --- | --- |
| **Financial Disclosure**  Enter a financial disclosure statement that describes the sources of funding for the work included in this submission. Review the [submission guidelines](http://journals.plos.org/plosntds/s/submission-guidelines#loc-financial-disclosure-statement) for detailed requirements. View published research articles from [*PLOS Neglected Tropical*](https://journals.plos.org/plosntds/) [*Diseases*](https://journals.plos.org/plosntds/) for specific examples.  This statement is required for submission and **will appear in the published article** if the submission is accepted. Please make sure it is accurate. | The author(s) received no specific funding for this work. |
| **Funded studies**  Enter a statement with the following details:   - Initials of the authors who received each award - Grant numbers awarded to each author - The full name of each funder - URL of each funder website - Did the sponsors or funders play any role in the study design, data collection and analysis, decision to publish, or preparation of the manuscript? |  |
| Did you receive funding for this work? |  |
| **Competing Interests**  On behalf of all authors, disclose any | The corresponding author has no competing interests. PE, MS, and KMS receive salaries from The Task Force for Global Health, an organization that receives funding from GSK and Johnson & Johnson, the manufacturers of albendazole and mebendazole, respectively |

| competing interests that could be perceived to bias this work.  This statement will be typeset if the manuscript is accepted for publication.  Review the instructions link below and PLOS NTDs’ [competing interests](https://journals.plos.org/plosntds/s/competing-interests#loc-what-represents-a-competing-interest) policy to determine what information must be disclosed at submission. |  |
| --- | --- |
| **Data Availability**  Provide a **Data Availability Statement** in the box below. This statement should detail where the data used in this submission can be accessed. This statement will be typeset if the manuscript is accepted for publication.  Before publication, authors are required to make all data underlying their findings fully available, without restriction. Review our [PLOS Data Policy](https://journals.plos.org/plosntds/s/data-availability) page for detailed information on this policy. Instructions for writing your Data Availability statement can be accessed via the Instructions link below. | The data used for the analyses in this paper are owned by the government of Kenya. Researchers are welcome to request access to de-identified datasets from the Division of Vector-Borne and Neglected Tropical Diseases at the Ministry of Health [(Wyckliff.omondi@gmail.com)](mailto:(Wyckliff.omondi@gmail.com) or through Children Without Worms [(cww@taskforce.org).](mailto:(cww@taskforce.org) |

manuscript	[Click here to access/download;Manuscript;1_Prevalence and](https://www2.cloud.editorialmanager.com/pntd/download.aspx?id=1346143&guid=70e17c21-f503-42a8-be4f-e446a2a08d93&scheme=1)[<https://www2.cloud.editorialmanager.com/pntd/download.aspx?id=1346143&guid=70e17c21-f503-42a8-be4f-e446a2a08d93&scheme=1> Intensity of STH in Narok and Bomet _Kenya.docx](https://www2.cloud.editorialmanager.com/pntd/download.aspx?id=1346143&guid=70e17c21-f503-42a8-be4f-e446a2a08d93&scheme=1)

1

2 **Prevalence and intensity of soil-transmitted helminth infections in Narok and Bomet**
3 **Counties, Kenya: Evidence from program monitoring**

4

5 Paul Kibati^1,2*^, Florence Wakesho^2^, Stephen Mwatha^1,2^, Denver Mariga^1,2^, Josephine Ihahi^1,2^,
6 Dickson Kioko^2^, Otieno Oloo^2^, Elvis Oyugi^1^, Sammy M. Njenga^3^, Ben Masiira^4^, Maurice
7 Owiny^1,4^, Cara Tupps^5^, Mariana Stephens^5^, Paul Emerson^5^, Wyckliff Omondi^2^, Rubina
8 Imtiaz^5^, Sultani Matendechero^6^, Sanjaya Dhakal^5^ and Kristin M. Sullivan^5^

9

10 ^1^*Field Epidemiology and Laboratory Training Program*, *Ministry of Health, Nairobi, Kenya*
11 ^2^*Division of Vector-Borne and Neglected Tropical Diseases, Ministry of Health, Nairobi,*
12 *Kenya*
13 *^3^Kenya Medical Research Institute, Nairobi, Kenya*
14 *^4^Africa Field Epidemiology Network, Kampala, Uganda*
15 *^5^Children Without Worms, The Task Force for Global Health, Decatur, Georgia, United*
16 *States of America*
17 *^6^National Public Health Institute, Nairobi, Kenya*

18

19

20

21

22 *[pgkibati@gmail.com](mailto:pgkibati@gmail.com)

# 23 Abstract

24 **Background**
25 The backbone of Kenya's soil-transmitted helminthiasis control program (Acronym if possible???)is the periodicdistribution of anti-helminthic Preventive Chemotherapy (PC). PC distribution has been
26 conducted since 2012 through the National School-Based Deworming Programme, which
27 deworms school-aged children (SAC, 5–14 years) living in areas at high risk for soil-
28 transmitted helminth (STH) infections. After nearly a decade of deworming, the Ministry of
29 Health sought to generate evidence for program monitoring by estimating the prevalence and
30 intensity of STH infections in Narok and Bomet counties among at-risk groups: preschool-
31 aged children (PSAC, 2–4 years), SAC, and women of reproductive age (WRA, 15–49 years).

# 32 Methods

33 During August and December of 2021, we conducted population-based household surveys in
34 Narok and Bomet counties, respectively, using a multi-stage, cluster random sample among
35 resident PSAC, SAC, and WRA. Individual and household questionnaires were administered
36 using an electronic mobile platform. Stool samples were collected and tested for roundworms
37 (*Ascaris lumbricoides*), hookworms (*Necator americanus, Ancylostoma duodenale*), and
38 whipworms (*Trichuris trichura*) using Kato-Katz method.

# 39 Results

40 Stool samples were provided by 1,062 PSAC, 1,922 SAC, and 364 WRA. Results indicated
41 that the prevalence of any STH infections was highest among SAC (16.2% [upper 95%
42 design-corrected confidence interval [U95% CI]: 23.1%]), followed by PSAC in Bomet
43 county (15.8% [U95% CI: 21.7%]). In Narok county, STHs prevalence was highest among
44 PSAC (12.8% [U95% CI: 18.2%]) followed by SAC (11.3% [U95% CI: 16.2%]). Moderate-
45 to-high intensity infection prevalence among PSAC and SAC exceeded the morbidity
46 elimination threshold of 2% in both counties. *A. lumbricoides* and *T. trichura* were most
47 identified; few hookworm infections were detected.

# 48 Conclusions

49 STH infections remain a public health problem in Narok and Bomet counties. There may be a
50 need to expand PC distribution to include risk groups beyond SAC during deworming
51 exercises, as envisioned in the Breaking Transmission Strategy, to hasten progress toward the
52 achievement of STH as a public health problem.

54

# 55 Author summary

56 Soil-transmitted helminth infections have been shown to affect children's nutrition and
57 cognitive development. Monitoring the prevalence of soil-transmitted helminth infections
58 among at-risk populations remains a key step in determining the type and frequency of
59 deworming medication to administer. Globally, treatment guidelines recommend treating
60 children and women of reproductive age, as they are at the highest risk of morbidity from
61 infection. In Kenya, preventive chemotherapy (PC) administration has primarily targeted
62 school-aged children using a school-based delivery platform. However, our program
63 monitoring survey demonstrated that the prevalence and intensity of STH infections among
64 PSAC and WRA continue to exceed the country’s elimination targets. The prevalence of any
65 STH infection was moderate (10-<20%) among children in both counties and low (2-<10%)
66 among WRA. This suggests the need to consider expanding PC administration to a
67 community-based model to make significant strides toward eliminating helminths as a public
68 health problem.

69

# 70 Introduction

71 Soil-transmitted helminth (STH) infections caused by roundworms (*Ascaris lumbricoides*),
72 hookworms (*Necator americanus*, *Ancylostoma duodenale*), and whipworms (*Trichuris*
73 *trichura*) are a public health problem globally, predominantly in the poorest regions of the
74 world [1,2]. The World Health Organization (WHO) classifies soil-transmitted helminthiasis
75 among the neglected tropical diseases (NTDs) and estimates that it affects more than 1.5
76 billion people globally, resulting in more than 12,000 deaths annually and 1.9 million
77 disability-adjusted life years [3,4].
78 STHs cause human infection through the ingestion of eggs from contaminated food
79 and soil or by active skin penetration in the case of hookworms [5]. STH infections are
80 known to cause both acute and chronic disease conditions such as anaemia, malnutrition,
81 abdominal pain and discomfort, diarrhoea, as well as impaired physical and cognitive
82 development [5,6]. WHO recommends controlling morbidity caused by STH infections
83 through preventive chemotherapy (PC) with the anthelmintic drugs albendazole or
84 mebendazole [7].
85 In Kenya, STH infections are widely distributed, occurring mainly in western and
86 coastal parts of the country, as well as other selected foci [5]. According to Kenya’s 2016-
87 2020 NTD strategic plan [(9)], it is estimated that 10 million people nationwide are affected
88 by STH, with children accounting for 50%. Another 16.6 million people are deemed to be at
89 risk of contracting STH infections [9–11]. The Ministry of Health, through the Division of
90 Vector-Borne and Neglected Tropical Diseases, launched the Breaking Transmission Strategy
91 in 2019, which aimed to eliminate morbidity due to STH infections [19]. This strategy seeks
92 to integrate interventions by focusing on increasing coverage of mass drug administration (MDA),
93 establishing sustainable implementation of necessary NTD-related water, sanitation, and
94 hygiene interventions, and implementing behaviour change communication interventions that
95 are necessary to move from control to elimination of STH infections as a public health
96 problem [13].
97 The launch of the Breaking Transmission Strategy was necessitated by the fact that
98 since 2012, PC has been provided by the National School-Based Deworming Programme
99 targeting school-aged children (SAC; 5-14 years) in various parts of the
100 country [14]. Within the first three years of implementation, the program demonstrated a
101 reduction of moderate-to-heavy mean intensity (MHI) of infection [14]. However, it was
102 noted that STH reinfections remained common following treatment, suggesting the need for
103 expanded interventions[15,16] and that, in areas of high prevalence, adults and children not in
104 school acted as reservoirs of STH, thereby impeding the effectiveness of STHs control
105 programs through school-based interventions only [17,18].
106 Given this concern, in 2021, the Ministry of Health Kenya, through the Division of
107 Vector-Borne and Neglected Tropical Diseases, sought to better understand the community-
108 wide prevalence of infection. They selected two counties, Narok and Bomet, where there had
109 been minimal reduction in STH prevalence among SAC, despite ongoing school-based
110 deworming efforts spanning over five years[15].

# 111 Objective

112 The objective of these community-based, cross-sectional surveys was to determine the
113 prevalence and intensity of STH infections among preschool-aged children (PSAC; 2–4
114 years), SAC (5–14 years), and women of reproductive age (WRA, 15–49 years) in
115 Narok and Bomet Counties.

# 116 Methods

117 **Ethics statement**
118 Ethical approval for the surveys was granted by the Ethics and Scientific Review Committee
119 of AMREF Health Africa in Kenya (P 705 – 2019). Permission to collect data was obtained
120 from the respective county/sub-county health offices. Participants and guardians were
121 provided with information about the purpose of the survey and what to expect. Written
122 informed consent by parents or guardians and verbal assent by participating children was
123 obtained before enrolment. What was the benefice for participants?

# 124 Survey setting and population

125 The surveys were conducted in Narok and Bomet counties (Fig. 1) in August and December
126 2021, respectively. Narok County covers an area of 17,933 km^2^ with a population of roughly
127 1.2 million. The county is administratively divided into six sub-counties and 30 wards, each
128 serving as smaller administrative sub-units of a sub-county[19]. Bomet County has an area of
129 2,037 km^2^ with a projected population of roughly 900,000. The county is divided into five
130 sub-counties and 25 wards[20]. PC has been distributed annually in these counties through
131 the National School-Based Deworming Programme since 2012.
132 **<<< FIG 1 >>>**

133

134

135

136

**Figure 1. Location of Bomet and Narok Counties, Kenya**

*Map prepared using shapefiles from OCHA Regional Office for Southern and Eastern Africa, Kenya Subnational Administrative Boundaries (retrieved from* [*https://data.humdata.org/dataset/cod-ab-kenon*](https://data.humdata.org/dataset/cod-ab-kenon) *on 28APR2022).*

# Survey design

137 We conducted a cross-sectional survey using the Integrated Community-based Survey for
138 Program Monitoring methodology. Full methodological details can be found in the online
139 reference manual (<https://childrenwithoutworms.org/icspm-reference-manual/>). Key details
140 are provided below.
141 The population-based household surveys were conducted using a multi-stage, cluster
142 random sampling technique, producing equal selection probabilities among resident PSAC,
143 SAC, and WRA in the respective counties. Within each county, population and housing data
144 from the Kenya National Bureau of Statistics, supplemented by county administrators, were
145 used to identify the villages[21]. Thirty villages/clusters were randomly selected from each
146 county in the first stage using population proportional to estimated size. The team selected
147 villages using the Survey Sample Builder developed by the Neglected Tropical Disease
148 Support Centre at the Task Force for Global Health ([https://childrenwithoutworms.org/icspm-](https://childrenwithoutworms.org/icspm-survey-sample-builder-tool/)
149 [survey-sample-builder-tool/](https://childrenwithoutworms.org/icspm-survey-sample-builder-tool/)).
150 The sample size for this survey was based on the transmission assessment survey
151 methodology published by WHO, which recommends at least 332 participants for each at-
152 risk group in a cluster survey [23]. To ensure the sample size was reached, 475 participants
153 were selected for inclusion for each risk group in each district to accommodate an
154 approximate non-response of 30%. Non-response was anticipated to cover both participants
155 who did not consent to be surveyed and those who did not provide stool samples. Households
156 were systematically selected using a pre-determined sampling interval, and the household
157 selection form aided field teams in knowing which risk group was to be sampled from the
158 household.

# 159 Recruitment and sample collection

160 Household members of selected households were included if they: a) were a member of the
161 selected risk groups of interest (PSAC, SAC, and WRA), b) slept at the household the night
162 before the interview, c) planned to stay at the household the night of the interview, d) were
163 able to provide a single stool sample within 24 hours of the interview, and e) were able and
164 willing to provide verbal consent or assent.
165 On the survey day, field officers visited the selected households and explained the
166 purpose of the survey and ensured consent/assent was obtained. The field teams were
167 comprised of a national-level supervisor from the Ministry of Health, a county-level
168 supervisor, a community health assistant (CHA) from the area, and a community health volunteer (CHV)
169 from the village being visited. Each participant was assigned a unique identification number,
170 which was used to track the collected sample and link it to the individual information
171 collected during the household visit. Participants were interviewed by trained data collectors
172 using a pre-tested, structured questionnaire. Socio-demographic information on the household
173 was provided by the household head/designate, and everyone provided their individual-level
174 information. Information for young children was provided by the household head/designate.
175 The participants were given a labelled wide-mouthed plastic container for stool collection, a
176 piece of plain paper, a piece of applicator sticks, and other sanitary necessities such as tissue
177 paper. The plastic containers were labelled with the participant's unique identification (ID) code
178 and their name, age, and sex to ensure a mix-up of containers didn’t occur within the
179 household. The explanation was given to the head of household or his designate if the
180 household head was absent. If the household head was not conversant in English, the
181 CHA explained in the local language. The field team explained safe
182 stool collection to the participant and asked them to wash their hands with soap and water
183 after providing samples. The fresh stool samples were collected the morning after the
184 household visit and interview.

185

186

# 187 Laboratory procedure

188 Stool samples were transported to the laboratory using cooler boxes within three hours of
189 collection. Stool samples were then prepared using a single thick smear technique using a
190 41.7 mg Kato-Katz template, with duplicate slides prepared and examined by two laboratory
191 Technicians under microscopes [23]. To maintain quality assurance, 10% of the slides were randomly selected andrexamined by a third technician blinded from the initial results from the other technicians.

# 192 Classification of infection intensity

193 The infection intensity, commonly called worm burden, is typically measured indirectly by
194 counting the number of eggs in faeces, expressed as eggs per gram (epg). This is because
195 indirect methods are more convenient and less invasive[24]. Direct methods of classifying
196 worm burden, which involve counting the number of expelled worms after anti-helminthic
197 treatment, may not be feasible during mass surveys and in areas with low worm burden.
198 WHO classifies infection intensity as light, moderate, or heavy using indirect methods for
199 each type of STH [24]. The epg estimate was applied to categorize the samples based on
200 infection intensity following WHO guidelines. For *A. lumbricoides* infections, light intensity
201 was defined as 1–4,999 epg, moderate intensity as 5,000–49,999 epg, and heavy intensity as
202 ≥50,000 epg. *T. trichiura* infections were classified as light intensity with 1–999 epg,
203 moderate intensity with 1,000–9,999 epg, and heavy intensity with ≥10,000 epg. For
204 hookworm infections, light intensity was defined as 1–1,999 epg, moderate intensity as
205 2,000–3,999 epg, and heavy intensity as ≥4,000 epg.

# 206 Data collection and management

207 Data were collected via mobile phone using Open Data Kit [ODK] and uploaded to a central
208 secure server. Three data sets [individual, household, and laboratory] were downloaded from
209 the server and saved on computers at the Ministry of Health Kenya. The data were cleaned,
210 and the household and individual files were merged with unique household ID
211 code and later with the laboratory file's unique individual ID code.

# 212 Statistical analyses and software

213 Descriptive statistics are presented using frequencies, proportions, and upper, one-sided 95%
214 confidence intervals (U95% CI) adjusted for sampling methodology. We used Microsoft
215 Excel, SAS, and R to manage and analyze the data.

# 216 Results

217 Characteristics of **survey population**
218 The survey teams visited sixty villages/clusters in the two counties, with Bomet and Narok
219 having 30 surveyed. However, due to an electronic data collection issue, participant
220 information from one village in Narok County was lost. A total of 3,348 individuals (M= ?and F=?) were
221 enrolled in the surveys, of whom 2,335 (69.7%) provided stool samples (Table 1).
222 Among the 2,096 children submitting a stool sample, 1,034 (49.3%) were female, and
223 the median age was 3 years for PSAC (interquartile range [IQR]:2–4), 9 years for SAC
224 (IQR:7–12), and 29 years for WRA (IQR: 20–40).
225 **Table 1. Number of clusters, households, and individuals surveyed by country and risk**
226 **group**
227 **<<< TABLE 1>>>**

# 228 Prevalence of infection

229 The prevalence of infections was slightly higher in Bomet County compared to Narok County
230 across all risk groups (Table 2 and Fig 2). Among children (PSAC and SAC), the estimated
231 prevalences ranged from 11% to 13% in Narok County and approximately 16% in Bomet
232 County. Among WRA, in both counties, the prevalence of any STH infections was less than
233 10%, with Narok at 6.6% and Bomet at 8.1%. In both counties, the prevalence of MHI
234 infection among children was above the 2% threshold required for elimination as a public
235 health problem. PSACs in Bomet County had a higher MHI infection prevalence among both
236 PSAC (6.1%) and SAC (5.5%) compared to corresponding risk groups in Narok County.
237 WRA in Bomet County recorded the lowest prevalence (0.3%) among all risk groups.

# 238 Parasite type

239 In both counties, *A. lumbricoides* caused the most infections, followed by *T. trichura.*
240 Hookworm infections were not detected in Bomet County and were uncommon in Narok
241 County. In Bomet County, the prevalence of *A. lumbricoides* infections was highest among
242 SAC, followed by PSAC, then WRA. In Narok County, *T. trichura* infections followed this
243 pattern; however, unlike Bomet County, the prevalence of *A. lumbridcoides* was highest
244 among PSAC rather than SAC.
245 **Table 2. Estimated prevalence of soil-transmitted helminth infections by county, species,**
246 **intensity, and risk group, Bomet and Narok Counties, 2021**
247 **<<< TABLE 2 >>>**
248 **<<< FIG 2 >>>**
249 **Fig 2. Estimated prevalence of soil-transmitted helminth infections by county, species,**
250 **intensity, and risk group, Bomet and Narok Counties, 2021**
251 *^*^N. americanus and A. duodenale*

# 252 Co-infections

253 Co-infections were uncommon (Table 3). When identified, they were most frequently
254 *A.lumbricoides* and *T.trichura,* with all risk categories in both counties recording at least one
255 co-infection. No triple infections were detected.
256 **Table 3. Estimated soil-transmitted helminth coinfection prevalence by county and risk**
257 **group**
258 **<<< TABLE 3 >>>**

# 259 Discussion

260 Our findings showed moderate (10-<20%) STH infections prevalence among children in
261 Bomet and Narok Counties. Prevalence estimates of any STH infections were above the 2%
262 treatment threshold target according to the breaking transmission strategy [12]. Additionally,
263 we found that MHI infection prevalence was above the WHO 2030 elimination as a public
264 health problem target of <2% among all surveyed children [7]. These findings suggest that
265 continued annual PC in these counties is required to make progress toward the elimination of
266 STH morbidity as a public health problem in the country.
267 Compared to prior surveys, the estimated prevalences among SAC from our
268 community-based surveys (Bomet, 16.2%, and Narok, 11.3%) were lower than those
269 demonstrated by school-based impact surveys conducted in 2017, five years after the
270 commencement of the National School-Based Deworming Programme. These 2017 surveys
271 showed a reduction of STH among SAC in Bomet from 29.7% in 2012 to 18.1% in 2017 and,
272 in Narok, from 53% in 2012 to 41% in 2017[15]. However, the prevalences are higher than
273 those derived from the ten-year model based geostatistical impact surveys (Bomet 8.3%, and
274 Narok 10.8%) conducted between 2021 and 2022 [25]. Despite there being an overall decline
275 in the prevalence of STHs from baseline in the country, the rate of decline in these two
276 counties has been relatively lower, which could be explained by poor sanitation facilities,
277 resulting in possible re-infection[26].

279

280

# 281 Parasite species

282 *A. lumbricoides* was the most common STH infections observed in the two counties, followed
283 by *T. trichura,* with very few hookworm infections detected. This finding corresponds with
284 school-based surveys conducted by the National School-based Deworming Programme,
285 which found *A. lumbricoides* to be the most common STHs during baseline, midline, and end-
286 line surveys in the country[15]. This may be due to helminths' long lifespan in human hosts
287 and their production of many environmentally resistant eggs, which are expelled in feces to
288 enhance their survival and lifecycle continuation, leading to reinfection [16,27]. The survey
289 also concurs with findings from the National School-based Deworming Programme that did
290 not show significant reductions in the prevalence of MHI infection with *T. trichura,* where
291 albendazole is the drug distributed. In contrast, it has been shown that a combination of
292 ivermectin and albendazole would be the drug of choice in areas with high *T. trichura*
293 infections[26]*.* It is also possible that reinfection occurs among the at-risk age groups,
294 resulting in a resurgence of prevalence, as stated in a meta-analysis that showed infection
295 after treatment occurs rapidly, particularly for *A. lumbricoides* and *T.trichura* [16]. Our
296 results suggest that the program should continue PC administration in these two counties to
297 reduce prevalence further.

# 298 Risk groups

299 Both infection prevalence and MHI infection prevalence were similar among PSAC and SAC
300 within both counties. The consistency in high prevalence among PSACs that are routinely
301 excluded from PC distributions highlights the fact that they should be targeted during
302 deworming exercises as infections among this age group lead to morbidity and cognitive
303 impairment and may even compromise the performance of PSAC on expressive
304 language [27]. In addition, given the prevalence of similar magnitude among PSAC as seen
305 among SAC, the Ministry may consider expanding beyond the current school-based PC
306 platform to the broader community. This expansion beyond SAC may enable the country to
307 accelerate progress toward eliminating STH as a public health problem in line with the
308 Breaking Transmission Strategy and the WHO 2030 NTD Roadmap.

# 309 Limitations

310 The survey was designed to estimate prevalence based on county-level administrative
311 boundaries. However, intra-county prevalence heterogeneity is likely. Hence, the program
312 would not be able to consider the diversity of the ecological conditions that have implications
313 on the drivers of STH transmission within the counties, contributing to the heterogeneous
314 classification of infection. This, in turn, would affect decision-making as to where PC
315 distribution would be most efficient and cost-effective for the program. Subsequently, with
316 these results, PC administration would be conducted in the entire county and not in specific
317 focal areas with a prevalence above 2%, contrary to the BTS which stipulates that the
318 implementation unit for treatment is the ward.
319 The sensitivity of the Kato Katz technique in detecting hookworm eggs is reduced in
320 low prevalence settings and with sample shipment delays between the time of collection in
321 the field and analysis in the laboratory. To mitigate this risk, laboratories were established at
322 the nearest health centres where teams were conducting the survey. This significantly reduced
323 the time between sample collection and laboratory analysis. Delays in sample shipment
324 would lead to an underestimation of the true prevalence of hookworm infections in the
325 sampled population[30,31].
326 During the survey conducted in Narok County, data from one village was lost,
327 reducing the sample size. This loss may have influenced the STH prevalence estimate in the
328 county. However, it's anticipated that any shifts in the county-level prevalence would likely
329 be minimal given that data from the missing village represent approximately 1/30th of the
330 total sample size.

331

# 332 Conclusion

333 Our survey showed that STH infections remain a public health problem in Narok and
334 Bomet counties. The elevated infection prevalence and MHI infection prevalence among all
335 surveyed groups, including PSAC and WRA, suggests that expansion to risk groups beyond
336 SAC during deworming exercises, as envisioned in the Breaking Transmission Strategy, may
337 accelerate progress to controlling STH infections.

# 338 Acknowledgments

339 We thank the children and parents who participated in this survey and the supervision
340 and technical support from the Division of Vector-Borne and Neglected Tropical Diseases,
341 Children Without Worms, and the Africa Field Epidemiology Network [AFENET]. We are
342 grateful to the field officers who collected the data and the data team who worked tirelessly to
343 ensure the survey was completed. We acknowledge the support from the national and county
344 management in Bomet and Narok counties.

345

346

347

348

# 349 References

350 1.	WHO. Ending the neglect to attain the Sustainable Development Goals: a road map for
351 neglected tropical diseases 2021–2030. Geneva: World Health Organization
352 (https://[www.who.int/neglected_diseases/Revised-DraftNTD-Roadmap-](http://www.who.int/neglected_diseases/Revised-DraftNTD-Roadmap-)
353 23Apr2020.pdf). Word Health Organization. 2020. 196 p.
354 2.	Id R, Mpanya A, Inocencio R, Id SL, Id EH, Boelaert M. Community-based survey on
355 helminth infections in Kwilu province , the Democratic Republic of the Congo , and
356 implications for local control strategies. 2020;1–14.
357 3.	Brooker S. Estimating the global distribution and disease burden of intestinal
358 nematode infections : Adding up the numbers – A review. Int J Parasitol [Internet].
359 2010;40(10):1137–44. Available from: <http://dx.doi.org/10.1016/j.ijpara.2010.04.004>
360 4.	Zeleke AJ, Derso A, Bayih AG, Gilleard JS, Eshetu T. Prevalence, Infection Intensity
361 and Associated Factors of Soil-Transmitted Helminthiasis Among School-Aged
362 Children from Selected Districts in Northwest Ethiopia. Res Rep Trop Med.
363 2021;Volume 12:15–23.
364 5.	Jourdan PM, Lamberton PHL, Fenwick A, Addiss DG. Seminar Soil-transmitted
365 helminth infections. Lancet [Internet]. 2017;6736(17):1–14. Available from:
366 <http://dx.doi.org/10.1016/S0140-6736(17)31930-X>
367 6.	Bethony J, Brooker S, Albonico M, Geiger SM, Loukas A, Diemert D, et al. Soil-
368 transmitted helminth infections : ascariasis , trichuriasis , and hookworm. 2006;367.
369 7.	World Health Organization (WHO). 2030 targets for soil-transmitted helminthiases
370 control programmes. 2012;
371 8.	Pullan RL, Gething PW, Smith JL, Mwandawiro CS, Sturrock HJW, Gitonga CW, et
372 al. Spatial Modelling of Soil-Transmitted Helminth Infections in Kenya : A Disease
373 Control Planning Tool. 2011;5(2).
374 9.	Ministry of Health Kenya. The 2nd Kenya National Strategic Plan For control of
375 NEGLECTED TROPICAL DISEASES 2016-2020. 2020;
376 10.	Ng’ang’a M, Matendechero S, Kariuki L, Omondi W, Makworo N, Owiti PO, et al.
377 Burden of Soil Transmitted Helminthiasis in Primary school children in Migori
378 County, Kenya. Suppl East African Med J. 2016;93(10):32–9.
379 11.	Ministry of Health Kenya. The 2nd Kenya National Strategic Plan For control of
380 Neglected Tropical Diseases. 2015.
381 12.	Ministry of Health Kenya. THE KENYA NATIONAL BREAKING
382 TRANSMISSION STRATEGY FOR SOIL-TRANSMITTED HELMINTHIASIS,
383 SCHISTOSOMIASIS, LYMPHATIC FILARIASIS AND TRACHOMA. 2019;
384 13.	Anderson RM, Turner HC, Truscott JE, Hollingsworth TD. Should the Goal for the
385 Treatment of Soil Transmitted Helminth ( STH ) Infections Be Changed from
386 Morbidity Control in Children to Community-Wide Transmission Elimination ?
387 2020;1–8.
388 14.	Okoyo C, Nikolay B, Kihara J, Simiyu E, Garn J V., Freeman MC, et al. Monitoring
389 the impact of a national school based deworming programme on soil-transmitted
390 helminths in Kenya: The first three years, 2012 - 2014. Parasites and Vectors
391 [Internet]. 2016;9(1):1–13. Available from: <http://dx.doi.org/10.1186/s13071-016->
392 1679-y
393 15.	Mwandawiro C, Okoyo C, Kihara J, Simiyu E, Kepha S, Campbell SJ, et al. Results of
394 a national school-based deworming programme on soil-transmitted helminths
395 infections and schistosomiasis in Kenya : 2012 – 2017. 2019;1–18.
396 16.	King CH, Zhou X nong, Jia T wu, Melville S. Soil-Transmitted Helminth Reinfection
397 after Drug Treatment : A Systematic Review and Meta-Analysis. 2012;6(5).
398 17.	Njenga SM, Mwandawiro CS, Muniu E, Mwanje MT, Haji FM, Bockarie MJ. Adult
399 population as potential reservoir of NTD infections in rural villages of Kwale district ,
400 Coastal Kenya : implications for preventive chemotherapy interventions policy.
401 2011;2–7.
402 18.	Njenga SM, Mutungi FM, Wamae CN, Mwanje MT, Njiru KK, Bockarie MJ. Once a
403 year school-based deworming with praziquantel and albendazole combination may not
404 be adequate for control of urogenital schistosomiasis and hookworm infection in
405 Matuga District , Kwale County , Kenya. 2014;1–9.
406 19.	Narok County Government. Narok County Integrated Development Plan 2018-2022.

407	2022;1–52.

408 20.	County Government of Bomet. County Government of Bomet County Integrated
409 Development Plan 2018-2022. 2018;(February 2018):518. Available from:
410 https://cog.go.ke/cog-reports/category/106-county-integrated-development-plans-
411 2018-2022?download=316:bomet-county-integrated-development-plan-2018-2022
412 21.	Kenya National Bureau of Statistics. Kenya Population and Housing Census 2019
413 Volume III [Internet]. Vol. 21. 2020. 1–9 p. Available from: [http://journal.um-](http://journal.um/)
414 surabaya.ac.id/index.php/JKM/article/view/2203
415 22.	World Health Organization (WHO). Assessing the Epidemiology of Soil Transmitted
416 Helminths during a Transmission Assessment Survey in the Global Programme for the
417 Elimination of Lymphatic Filariasis. 2015.
418 23.	Katz N, Chaves A, Pellegrino J. A simple device for quantitative stool thick-smear
419 technique in Schistosomiasis mansoni. Rev Inst Med Trop Sao Paulo. 1972;14(6):397–
420 400.
421 24.	World Health Organization (WHO). Preventive chemotherapy guideline to control
422 soil-transmitted helminth infections in at-risk population groups. 2017.
423 25.	Okoyo C, Minnery M, Orowe I, Owaga C, Wambugu C, Olick N, et al. Using a model-
424 based geostatistical approach to design and analyse the prevalence of schistosomiasis
425 in Kenya. 2023;(November):1–10.
426 26.	Masaku J, Okoyo C, Araka S, Musuva R, Njambi E, Njomo DW, et al. PLOS
427 NEGLECTED TROPICAL DISEASES Understanding factors responsible for the slow
428 decline of soil-transmitted helminthiasis following seven rounds of annual mass drug
429 administration ( 2012 – 2018 ) among school children in endemic counties of Kenya :
430 A mixe. 2023;1–29. Available from: <http://dx.doi.org/10.1371/journal.pntd.0011310>
431 27.	Flammer PG, Smith AL, Smith AL. Intestinal helminths as a biomolecular complex in
432 archaeological research. 2020;
433 28.	Hürlimann E, Keller L, Patel C, Welsche S, Hattendorf J, Ali SM, et al. Efficacy and
434 safety of co-administered ivermectin and albendazole in school-aged children and
435 adults infected with Trichuris trichiura in Côte d’Ivoire, Laos, and Pemba Island,
436 Tanzania: a double-blind, parallel-group, phase 3, randomised controlled trial. Lancet
437 Infect Dis. 2022;22(1):123–35.
438 29.	Mazibuko XI, Chimbari M. The effect of schistosomiasis and soil-transmitted
439 helminths on expressive language skills among African preschool children. BMC
440 Infect Dis [Internet]. 2022;22(1):1–9. Available from: https://doi.org/10.1186/s12879-
441 022-07260-2
442 30.	Loukas A, Hotez PJ, Diemert D, Yazdanbakhsh M, Correa-oliveira R, Croese J.
443 Hookworm infection. 2016;

| 444 31. | Bosch F, Palmeirim MS, Ali SM, Ame SM, Hattendorf J, Keiser J, et al. Diagnosis of |
| --- | --- |
| 445 | soil-transmitted helminths using the kato-katz technique: What is the influence of |
| 446 | stirring, storage time and storage temperature on stool sample egg counts? PLoS Negl |
| 447 | Trop Dis [Internet]. 2021;15(1):1–17. Available from: |
| 448 | <http://dx.doi.org/10.1371/journal.pntd.0009032> |
| 449 |  |

Figure_Map of Kenya	[Click here to access/download;Figure;2_Fig 1 - Map of Kenya.png](https://www2.cloud.editorialmanager.com/pntd/download.aspx?id=1346145&guid=4207e24e-4246-4cc5-aeef-1fe565960c80&scheme=1)[<https://www2.cloud.editorialmanager.com/pntd/download.aspx?id=1346145&guid=4207e24e-4246-4cc5-aeef-1fe565960c80&scheme=1>](https://www2.cloud.editorialmanager.com/pntd/download.aspx?id=1346145&guid=4207e24e-4246-4cc5-aeef-1fe565960c80&scheme=1)

Figure	[Click here to access/download;Figure;Fig 2 STH prevalence by risk group.pdf](https://www2.cloud.editorialmanager.com/pntd/download.aspx?id=1347768&guid=8eba811e-84fb-43f4-9b8e-c205505c8ab7&scheme=1)[<https://www2.cloud.editorialmanager.com/pntd/download.aspx?id=1347768&guid=8eba811e-84fb-43f4-9b8e-c205505c8ab7&scheme=1>](https://www2.cloud.editorialmanager.com/pntd/download.aspx?id=1347768&guid=8eba811e-84fb-43f4-9b8e-c205505c8ab7&scheme=1)

13:53 Friday, September 16, 2022	**1**

**Kenya** **-** **Prevalence** **of** **any** **STH** **infection** **and** **moderate** **or** **heavy** **intensity** **(MHI)** **STH** **infection** **by**

**district** **and** **risk** **group**

30

Bomet

Narok

15.8	6.1

16.2	5.5

8.1	0.7

12.8	3.3

11.3	2.7

6.6	3.3

0.0	0.0

0.0	0.0

0.0	0.0

0.3	0.0

0.3	0.0

1.1	0.0

10.8	5.8

11.3	5.5

6.1	0.7

9.5	2.7

6.3	2.4

4.4	3.3

6.4	0.8

8.4	0.1

4.1	0.0

4.6	1.1

6.8	0.5

2.2	0.0

20

Any STH

10

0

30

20

Hookworm

10

**Prevalence** **of** **infection**

0

30

20

Ascaris

10

0

30

20

Trichuris

10

0

PSAC	SAC	WRA

**Risk** **group**

PSAC	SAC	WRA

*Error* *bars* *represent* *the* *upper* *95%* *confidence* *limit.*

Any infection

MHI infection

*Run* *with* *program:* *D_Create_Prevalence_Plots_16SEP2022* *on* *16SEP22.* *File* *used:* *15SEP2022* *prg* *dt\15SEP22* *run* *dt\Results* *-* *All* *Districts.xlsx.*

Table1_survey population	[Click here to access/download;Table;3_Table 1 - Survey](https://www2.cloud.editorialmanager.com/pntd/download.aspx?id=1346146&guid=de76622c-1091-46d5-ad6e-b79e4707398c&scheme=1)[<https://www2.cloud.editorialmanager.com/pntd/download.aspx?id=1346146&guid=de76622c-1091-46d5-ad6e-b79e4707398c&scheme=1> Population.docx](https://www2.cloud.editorialmanager.com/pntd/download.aspx?id=1346146&guid=de76622c-1091-46d5-ad6e-b79e4707398c&scheme=1)

**Table 1. Number of clusters, households, and individuals surveyed by county and risk group**

| **County** | **Clusters^*^** | | **Households** | | **Individuals** | | | |
| --- | --- | --- | --- | --- | --- | --- | --- | --- |
|  | **Sur- veyed**  **n** | **House- holds per cluster**  **mean (SD)** | **En- rolled**  **n** | **Enrolled households where ≥ 1 member submitted a stool sample**  **n (%)** | **Risk group** | **En- rolled**  **n** | **Among those enrolled, number of individuals submitting stool sample**  **n (%)** | **Among those submitting stool sample, number female**  **n (%)** |
| **Bomet** | 30 | 21.8  (6.0) | 654 | 530 (81.0%) | **PSAC** | 508 | 361 (71.1%) | 186 (51.5%) |
|  |  |  |  |  | **SAC** | 1,014 | 749 (73.9%) | 387 (51.7%) |
|  |  |  |  |  | **WRA** | 225 | 148 (65.8%) | 148 (100.0%) |
| **Narok** | 29^†^ | 18.9  (6.3) | 547 | 429 (78.4%) | **PSAC** | 554 | 367 (66.2%) | 186 (50.1%) |
|  |  |  |  |  | **SAC** | 908 | 619 (68.2%) | 275 (44.4%) |
|  |  |  |  |  | **WRA** | 139 | 91 (65.5%) | 91 (100.0%) |

*Abbreviations: n–Count; PSAC–preschool-aged children (2–4 years old); SAC–school-aged children (5–14 years old); SD–Standard Deviation; WRA–women of reproductive age (15–49 years old)*

*^*^Clusters are typically a single village; however, in some cases, villages are combined if the projected village size in households is less than the target segment size.*

*^†^Information from surveys in one cluster was lost due to an electronic data collection issue. Therefore, only information on 29 clusters was included in the analysis.*

Table 2_Results	[Click here to access/download;Table;4_Table 2 - Results.docx](https://www2.cloud.editorialmanager.com/pntd/download.aspx?id=1346147&guid=c9b812de-38ed-471f-b0d9-d6892e0ad644&scheme=1)[<https://www2.cloud.editorialmanager.com/pntd/download.aspx?id=1346147&guid=c9b812de-38ed-471f-b0d9-d6892e0ad644&scheme=1>](https://www2.cloud.editorialmanager.com/pntd/download.aspx?id=1346147&guid=c9b812de-38ed-471f-b0d9-d6892e0ad644&scheme=1)

**Table 2. Estimated prevalence of soil-transmitted helminth infection by county, species, intensity, and risk group, Bomet and Narok Counties, 2021**

| **County** | **Risk group** | **Submitting stool samples**  **n** | **Hookworm^*^**  **p (95% UCL)** | ***A. lumbricoides***  **p (95% UCL)** | ***T. trichiura***  **p (95% UCL)** | **Any STH**  **p (95% UCL)** |
| --- | --- | --- | --- | --- | --- | --- |
| **Any intensity infections** | | | | | | |
| **Bomet** | **PSAC** | 361 | 0.0% | 10.8% (16.1%) | 6.4% (10.3%) | 15.8% (21.7%) |
|  | **SAC** | 749 | 0.0% | 11.3% (15.9%) | 8.4% (15.2%) | 16.2% (23.1%) |
|  | **WRA** | 148 | 0.0% | 6.1% (11.9%) | 4.1% (9.4%) | 8.1% (14.5%) |
| **Narok** | **PSAC** | 367 | 0.3% (1.2%) | 9.5% (14.5%) | 4.6% (7.2%) | 12.8% (18.2%) |
|  | **SAC** | 619 | 0.3% (1.5%) | 6.3% (10.0%) | 6.8% (10.7%) | 11.3% (16.2%) |
|  | **WRA** | 91 | 1.1 (5.1%) | 4.4% (10.8%) | 2.2% (6.7%) | 6.6% (13.3%) |
| **Moderate-to-heavy intensity infections** | | | | | | |
| **Bomet** | **PSAC** | 361 | 0.0% | 5.8% (9.3%) | 0.8% (2.1%) | 6.1% (9.5%) |
|  | **SAC** | 749 | 0.0% | 5.5% (8.7%) | 0.1% (0.6%) | 5.5% (8.7%) |
|  | **WRA** | 148 | 0.0% | 0.7% (3.2%) | 0.0% | 0.7% (3.2%) |
| **Narok** | **PSAC** | 367 | 0.0% | 2.7% (5.7%) | 1.1% (2.9%) | 3.3% (6.3%) |
|  | **SAC** | 619 | 0.0% | 2.4% (4.3%) | 0.5% (1.6%) | 2.7% (4.7%) |
|  | **WRA** | 91 | 0.0% | 3.3% (8.0%) | 0.0% | 3.3% (8.0%) |

*Abbreviations: n–Count; p–Prevalence; UCL–Upper 95% one-sided confidence limit; PSAC–preschool-aged children (2–4 years old); SAC–school-aged children (5–14 years old); STH–Soil-transmitted helminth; WRA–women of reproductive age (15–49 years old)*

*^*^N. americanus and A. duodenale*

Table 3_coinfections	[Click here to access/download;Table;6_Table 3 - Coinfections.docx](https://www2.cloud.editorialmanager.com/pntd/download.aspx?id=1346148&guid=92f79c59-5558-4910-ae52-7f7fdf5e7a43&scheme=1)[<https://www2.cloud.editorialmanager.com/pntd/download.aspx?id=1346148&guid=92f79c59-5558-4910-ae52-7f7fdf5e7a43&scheme=1>](https://www2.cloud.editorialmanager.com/pntd/download.aspx?id=1346148&guid=92f79c59-5558-4910-ae52-7f7fdf5e7a43&scheme=1)

**Table 3. Estimated soil-transmitted helminth coinfection prevalence by county and risk group**

| **County** | **Risk group** | **Sub- mitting stool samples**  **n** | **Uninfected**  **n (p)** | **Mono- infection** | | | **Co-infection** | | | **Triple infection** |
| --- | --- | --- | --- | --- | --- | --- | --- | --- | --- | --- |
|  |  |  |  | **Hookworm^*^**  **n (p)** | ***A. lumbri- coides***  **n (p)** | ***T. trichiura***  **n (p)** | **Hookworm and**  ***A. lumbri- coides***  **n (p)** | **Hookworm and**  ***T. trichiura***  **n (p)** | ***A. lumbri- coides* and**  ***T. trichiura***  **n (p)** | **Hookworm, *A. lumbricoides*, and *T. trichiura***  **n (p)** |
| **Bomet** | **PSAC** | 361 | 304 (84.2%) | 0 (0.0%) | 34 (9.4%) | 18 (5%) | 0 (0.0%) | 0 (0.0%) | 5 (1.4%) | 0 (0.0%) |
|  | **SAC** | 749 | 628 (83.8%) | 0 (0.0%) | 58 (7.7%) | 36 (4.8%) | 0 (0.0%) | 0 (0.0%) | 27 (3.6%) | 0 (0.0%) |
|  | **WRA** | 148 | 136 (91.9%) | 0 (0.0%) | 6 (4.1%) | 3 (2.0%) | 0 (0.0%) | 0 (0.0%) | 3 (2.0%) | 0 (0.0%) |
| **Narok** | **PSAC** | 367 | 320 (87.2%) | 0 (0.0%) | 29 (7.9%) | 12 (3.3%) | 1 (0.3%) | 0 (0.0%) | 5 (1.4%) | 0 (0.0%) |
|  | **SAC** | 619 | 549 (88.7%) | 1 (0.2%) | 27 (4.4%) | 29 (4.7%) | 0 (0.0%) | 1 (0.2%) | 12 (1.9%) | 0 (0.0%) |
|  | **WRA** | 91 | 85 (93.4%) | 1 (1.1%) | 3 (3.3%) | 1 (1.1%) | 0 (0.0%) | 0 (0.0%) | 1 (1.1%) | 0 (0.0%) |

*Abbreviations: n–Count; p–Prevalence; PSAC–preschool-aged children (1–4 years old); SAC–school-aged children (5–14 years old); WRA–women of reproductive age (15–49 years old)*

*^*^N. americanus and A. duodenale*

Contributions

[Click here to access/download](https://www2.cloud.editorialmanager.com/pntd/download.aspx?id=1346149&guid=5ca31beb-5feb-4216-a5c8-b624df32aa05&scheme=1)

[**Supporting Information**](https://www2.cloud.editorialmanager.com/pntd/download.aspx?id=1346149&guid=5ca31beb-5feb-4216-a5c8-b624df32aa05&scheme=1)

[Other info needed to submit_31JULY24.docx](https://www2.cloud.editorialmanager.com/pntd/download.aspx?id=1346149&guid=5ca31beb-5feb-4216-a5c8-b624df32aa05&scheme=1)
