# Supplementary material for: Prevalence and intensity of soil-transmitted helminth infections in Narok and Bomet Counties, Kenya: Evidence from program monitoring
Source: PLoS Negl Trop Dis. 2025 Dec 30;19(12):e0012415. doi: 10.1371/journal.pntd.0012415 (PMC12753061; doi:10.1371/journal.pntd.0012415)
Supplement: Attachment — Submitted filename: Comments_MS_PLOS-NTD-24-01087.docx [file pntd.0012415.s003.docx]

**COMMENTS**

***General comments***

This work is topical and of interest in public health, in the prevention and elimination of neglected tropical diseases, and in particular intestinal parasitic infections. This work will help to provide essential, even vital, information for the management of populations in environments that continue to pay a heavy price for these parasitic affections.

**Strength:** This work is part of the implementation of the World Health Organization (WHO) 2020-2030 roadmap;

-From controlling morbidity to eliminating neglected tropical diseases.

**Weakness:** Low spectrum of intervention, given that this is national STH prevention

***Methods***

***Ethical Consideration***

Wouldn't there be a National Ethics Committee in Kenya for ethical approval?

Were children aged 2-4 able to give verbal consent? What was the benefit for participants, particularly those diagnosed as positive?

***Survey setting and population***

Why did you choose particularly Narok and Bomet for this study? Is there an explanation?

What were the criteria used to select the composite locations?

The methods section is poorly structured!

***Statistical analysis***

The statistical analysis section is not rich in information. No statistical tests have been applied! I suggest that you carry out comparative analyses by gender, age group (intra county) and between counties.

What did you do with the questionnaire data? Wasn't it possible to highlight risk factors?

***Results***

The results do not follow the objectives set, but they are confused!

From the above, I propose the following plan:

i) Parasites infections;

ii) Intensity of infection;

iii) Parasites infection status, stratified by age and sex

iv) Co-infections probably;

v) Associations between parasites infections and sociodemographic factors.

**Discussion**

- The discussion is descriptive rather than explanatory.

- The main outcome (effect of interventions on parasitic infections) is poorly documented.

-Page 14 line 295-297: more a recommendation than a discussion

The discussion explains the key results announced in the results section.

**The other comments were mentioned in the document**.
