# Supplementary material for: Prevalence and intensity of soil-transmitted helminth infections in Narok and Bomet Counties, Kenya: Evidence from program monitoring
Source: PLoS Negl Trop Dis. 2025 Dec 30;19(12):e0012415. doi: 10.1371/journal.pntd.0012415 (PMC12753061; doi:10.1371/journal.pntd.0012415)
Supplement: Attachment — Submitted filename: Kibati_et_al_2024_revision.docx [file pntd.0012415.s004.docx]

**Prevalence and intensity of soil-transmitted helminth infections in Narok and Bomet Counties, Kenya: Evidence from program monitoring**

Paul Kibati^1,2*^, Florence Wakesho^2^, Stephen Mwatha^1,2^, Denver Mariga^1,2^, Josephine Ihahi^1,2^, Dickson Kioko^2^, Otieno Oloo^2^, Elvis Oyugi^1^, Sammy M. Njenga^3^, Ben Masiira^4^, Maurice Owiny^1,4^, Cara Tupps^5^, Mariana Stephens^5^, Paul Emerson^5^, Wyckliff Omondi^2^, Rubina Imtiaz^5^, Sultani Matendechero^6^, Sanjaya Dhakal^5^ and Kristin M. Sullivan^5^

^1^*Field Epidemiology and Laboratory Training Program*, *Ministry of Health, Nairobi, Kenya*

^2^*Division of Vector-Borne and Neglected Tropical Diseases, Ministry of Health, Nairobi, Kenya*

*^3^Kenya Medical Research Institute, Nairobi, Kenya*

*^4^Africa Field Epidemiology Network, Kampala, Uganda*

*^5^Children Without Worms, The Task Force for Global Health, Decatur, Georgia, United States of America*

*^6^National Public Health Institute, Nairobi, Kenya*

*[pgkibati@gmail.com](mailto:pgkibati@gmail.com)

# Abstract

Background

The backbone of Kenya's soil-transmitted helminthiasis control program is the periodic distribution of anti-helminthic Preventive Chemotherapy (PC). PC distribution has been conducted since 2012 through the National School-Based Deworming Programme, which deworms school-age children (SAC, 5–14 years) living in areas at high risk for soil-transmitted helminth (STH) infections. After nearly a decade of deworming, the Ministry of Health sought to generate evidence for program monitoring by estimating the prevalence and intensity of STH infection in Narok and Bomet counties among at-risk groups: preschool-aged children (PSAC, 2–4 years), SAC, and women of reproductive age (WRA, 15–49 years).

Methods

During August and December of 2021, we conducted population-based household surveys in Narok and Bomet counties, respectively, using a multi-stage, cluster random sample among resident PSAC, SAC, and WRA. Individual and household questionnaires were administered using an electronic mobile platform. Stool samples were collected and tested for roundworms (*Ascaris lumbricoides*), hookworms (*Necator americanus, Ancylostoma duodenale*), and whipworms (*Trichuris trichura*) using duplicate thick-smear Kato-Katz microscopy.

Results

Stool samples were provided by 1,062 PSAC, 1,922 SAC, and 364 WRA. Results indicated that the prevalence of any STH infection was highest among SAC (16.2% [upper 95% design-corrected confidence interval [U95% CI]: 23.1%]), followed by PSAC in Bomet county (15.8% [U95% CI: 21.7%]). In Narok county, STH prevalence was highest among PSAC (12.8% [U95% CI: 18.2%]) followed by SAC (11.3% [U95% CI: 16.2%]). Moderate-to-high intensity infection prevalence among PSAC and SAC exceeded the morbidity elimination threshold of 2% in both counties. *A. lumbricoides* and *T. trichura* were most identified; few hookworm infections were detected.

Conclusions

STH infections remain a public health problem in Narok and Bomet counties. There may be a need to expand PC distribution to include risk groups beyond SAC during deworming exercises, as envisioned in the Breaking Transmission Strategy, to hasten progress toward the achievement of STH as a public health problem.

# Author summary

Soil-transmitted helminth infections have been shown to affect children's nutrition and cognitive development. Monitoring the prevalence of soil-transmitted helminth infections among at-risk populations remains a key step in determining the type and frequency of deworming medication to administer. Globally, treatment guidelines recommend treating children and women of reproductive age, as they are at the highest risk of morbidity from infection. In Kenya, preventive chemotherapy (PC) administration has primarily targeted school-aged children using a school-based delivery platform. However, our program monitoring survey demonstrated that the prevalence and intensity of STH infections among PSAC and WRA continue to exceed the country’s elimination targets. The prevalence of any STH infection was moderate (10-<20%) among children in both counties and low (2-<10%) among WRA. This suggests the need to consider expanding PC administration to a community-based model to make significant strides toward eliminating helminths as a public health problem.

# Introduction

Soil-transmitted helminth (STH) infections caused by roundworms (*Ascaris lumbricoides*), hookworms (*Necator americanus*, *Ancylostoma duodenale*), and whipworms (*Trichuris trichura*) are a public health problem globally, predominantly in the poorest regions of the world [1,2]. The World Health Organization (WHO) classifies soil-transmitted helminthiasis among the neglected tropical diseases (NTDs) and estimates that it affects more than 1.5 billion people globally, resulting in more than 12,000 deaths annually and 1.9 million disability-adjusted years [3,4].

STHs cause human infection through the ingestion of eggs from contaminated food and soil or by active skin penetration in the case of hookworms [5]. STH infections are known to cause both acute and chronic disease conditions such as anaemia, malnutrition, abdominal pain and discomfort, diarrhoea, as well as impaired physical and cognitive development [5,6]. WHO recommends controlling morbidity caused by STH infections through preventive chemotherapy (PC) with the anthelmintic drugs albendazole or mebendazole [7].

In Kenya, STH infections are widely distributed, occurring mainly in western and coastal parts of the country, as well as other selected foci [5]. According to Kenya’s 2016-2020 NTD strategic plan [(9)], it is estimated that 10 million people nationwide are affected by STH, with children accounting for 50%. Another 16.6 million people are deemed to be at risk of contracting STH infections [9–11]. The Ministry of Health, through the Division of Vector-Borne and Neglected Tropical Diseases, launched the Breaking Transmission Strategy in 2019, which aimed to eliminate morbidity due to STH infections [19]. This strategy seeks to integrate interventions by focusing on increasing coverage of mass drug administration, establishing sustainable implementation of necessary NTD-related water, sanitation, and hygiene interventions, and implementing behaviour change communication interventions that are necessary to move from control to elimination of STH infections as a public health problem [13].

The launch of the Breaking Transmission Strategy was necessitated by the fact that since 2012, PC has been provided by the National School-Based Deworming Programme targeting school-aged children (SAC) between 5 and 14 years of age in various parts of the country [14]. Within the first three years of implementation, the program demonstrated a reduction of moderate-to-heavy (MHI) mean intensity of infection [14]. However, it was noted that STH reinfections remained common following treatment, suggesting the need for expanded interventions [15,16] and that, in areas of high prevalence, adults and children not in school acted as reservoirs of STH, thereby impeding the effectiveness of STH control programs through school-based interventions only [17,18].

Given this concern, in 2021, the Ministry of Health Kenya, through the Division of Vector-Borne and Neglected Tropical Diseases, sought to better understand the community-wide prevalence of infection. They selected two counties, Narok and Bomet, where there had been minimal reduction in STH prevalence among SAC, despite ongoing school-based deworming efforts spanning over five years [15].

**Objective**

The objective of these community-based, cross-sectional surveys was to determine the prevalence and intensity of STH infections among preschool-aged children (PSAC, ages 2–4 years), SAC (ages 5–14 years), and women of reproductive age (WRA, 15–49 years) in Narok County and Bomet County.

# Methods

Ethics statement

Ethical approval for the surveys was granted by the Ethics and Scientific Review Committee of AMREF Health Africa in Kenya (P 705 – 2019). Permission to collect data was obtained from the respective county/sub-county health offices. Participants and guardians were provided with information about the purpose of the survey and what to expect. Written informed consent by parents or guardians and verbal assent by participating children was obtained before enrolment.

Survey setting and population

The surveys were conducted in Narok and Bomet counties (Fig. 1) in August and December 2021, respectively. Narok County covers an area of 17,933 km^2^ with a population of roughly 1.2 million. The county is administratively divided into six sub-counties and 30 wards, each serving as smaller administrative sub-units of a sub-county[19]. Bomet County has an area of 2,037 km^2^ with a projected population of roughly 900,000. The county is divided into five sub-counties and 25 wards[20]. PC has been distributed annually in these counties through the National School-Based Deworming Programme since 2012.

**<<< FIG 1 >>>**

**Figure 1. Location of Bomet and Narok Counties, Kenya**

*Map prepared using shapefiles from OCHA Regional Office for Southern and Eastern Africa, Kenya Subnational Administrative Boundaries (retrieved from* [*https://data.humdata.org/dataset/cod-ab-kenon*](https://data.humdata.org/dataset/cod-ab-kenon) *on 28APR2022).*

Survey design

We conducted a cross-sectional survey using the Integrated Community-based Survey for Program Monitoring methodology. Full methodological details can be found in the online reference manual (<https://childrenwithoutworms.org/icspm-reference-manual/>). Key details are provided below.

The population-based household surveys were conducted using a multi-stage, cluster random sampling technique, producing equal selection probabilities among resident PSAC, SAC, and WRA in the respective counties. Within each county, population and housing data from the Kenya National Bureau of Statistics, supplemented by county administrators, were used to identify the villages[21]. Thirty villages/clusters were randomly selected from each county in the first stage using population proportional to estimated size. The team selected villages using the Survey Sample Builder developed by the Neglected Tropical Disease Support Centre at the Task Force for Global Health (<https://childrenwithoutworms.org/icspm-survey-sample-builder-tool/>).

The sample size for this survey was based on the transmission assessment survey methodology published by WHO, which recommends at least 332 participants for each at-risk group in a cluster survey [23]. To ensure the sample size was reached, 475 participants were selected for inclusion for each risk group in each district to accommodate an approximate non-response of 30%. Non-response was anticipated to cover both participants who did not consent to be surveyed and those who did not provide stool samples. Households were systematically selected using a pre-determined sampling interval, and the household selection form aided field teams in knowing which risk group was to be sampled from the household.

Recruitment and sample collection

Household members of selected households were included if they: a) were members of the selected risk groups of interest (PSAC, SAC, and WRA), b) slept at the household the night before the interview, c) planned to stay at the household the night of the interview, d) were able to provide a single stool sample within 24 hours of the interview, and e) were able and willing to provide verbal consent or assent.

On the survey day, field officers visited the selected households, explained the purpose of the survey and ensured consent/assent was obtained. The field teams were comprised of a national-level supervisor from the Ministry of Health, a county-level supervisor, a community health assistant from the area, and a community health volunteer from the village being visited. Each participant was assigned a unique identification number, which was used to track the collected sample and link it to the individual information collected during the household visit. Participants were interviewed by trained data collectors using a pre-tested, structured questionnaire. Socio-demographic information on the household was provided by the household head/designate, and everyone provided their individual-level information. Information for young children was provided by the household head/designate.

The participants were given a labelled wide-mouthed container for stool collection, a piece of plain paper, a piece of applicator sticks, and other sanitary necessities such as tissue paper. The plastic containers were labelled with the participants' unique identification numbers and their name, age, and sex to ensure a mix-up of containers did not occur within the household. The explanation was given to the head of household or his designate if the household head was absent. If the household head was not conversant in English, the community health assistant explained in the local language. The field team explained safe stool collection to the participant and asked them to wash their hands with soap and water after providing samples. The fresh stool samples were collected the morning after the household visit and interview.

Laboratory methods

Stool samples were transported to the laboratory using cooler boxes within three hours of collection. Stool samples were then prepared using a single thick smear technique using a 41.7 mg Kato-Katz template, with duplicate slides prepared and read by two laboratory technicians[23]. To maintain quality assurance, 10% of the slides were randomly selected and read by a third technician blinded from the initial results from the other technicians.

Classification of infection intensity

The infection intensity, commonly called worm burden, is typically measured indirectly by counting the number of eggs in faeces, expressed as eggs per gram (epg). This is because indirect methods are more convenient and less invasive[24]. Direct methods of classifying worm burden, which involve counting the number of expelled worms after anti-helminthic treatment, may not be feasible during mass surveys and in areas with low worm burden. WHO classifies infection intensity as light, moderate, or heavy using indirect methods for each type of STH [24]. The epg estimate was applied to categorize the samples based on infection intensity following WHO guidelines. For *A. lumbricoides* infections, light intensity was defined as 1–4,999 epg, moderate intensity as 5,000–49,999 epg, and heavy intensity as ≥50,000 epg. *T. trichiura* infections were classified as light intensity with 1–999 epg, moderate intensity with 1,000–9,999 epg, and heavy intensity with ≥10,000 epg. For hookworm infections, light intensity was defined as 1–1,999 epg, moderate intensity as 2,000–3,999 epg, and heavy intensity as ≥4,000 epg.

Data collection and management

Data were collected via mobile phone using Open Data Kit [ODK] and uploaded to a central secure server. Three data sets [individual, household, and laboratory] were downloaded from the server and saved on computers at the Ministry of Health Kenya. The data were cleaned, and the household and individual files were merged with unique household identification numbers and later with the laboratory file's unique individual identification numbers.

Statistical analyses and software

Descriptive statistics are presented using frequencies, proportions, and upper, one-sided 95% confidence intervals (U95% CI) adjusted for sampling methodology. We used Microsoft Excel, SAS, and R to manage and analyze the data.

# Results

Survey population

The survey teams visited sixty villages/clusters in the two counties, with Bomet and Narok having 30 surveyed. However, due to an electronic data collection issue, participant information from one village in Narok County was lost. A total of 3,348 individuals were enrolled in the surveys, of whom 2,335 (69.7%) provided stool samples (Table 1).

Among the 2,096 children submitting a stool sample, 1,034 (49.3%) were female, and the median age was 3 years for PSAC (interquartile range [IQR]:2–4), 9 years for SAC (IQR:7–12), and 29 years for WRA (IQR: 20–40).

**Table 1. Number of clusters, households, and individuals surveyed by country and risk group**

**<<< TABLE 1>>>**

Prevalence of infection

The prevalence of infections was slightly higher in Bomet County compared to Narok County across all risk groups (Table 2 and Fig 2). Among children (PSAC and SAC), the estimated prevalences ranged from 11% to 13% in Narok County and approximately 16% in Bomet County. Among WRA, in both counties, the prevalence of any STH infection was less than 10%, with Narok at 6.6% and Bomet at 8.1%. In both counties, the prevalence of MHI infection among children was above the 2% threshold required for elimination as a public health problem. PSACs in Bomet County had a higher MHI infection prevalence among both PSAC (6.1%) and SAC (5.5%) compared to corresponding risk groups in Narok County. WRA in Bomet County recorded the lowest prevalence (0.3%) among all risk groups.

Parasite type

In both counties, *A. lumbricoides* caused the most infections, followed by *T. trichura.* Hookworm infections were not detected in Bomet County and were uncommon in Narok County. In Bomet County, the prevalence of *A. lumbricoides* infections was highest among SAC, followed by PSAC, then WRA. In Narok County, *T. trichura* infections followed this pattern; however, unlike Bomet County, the prevalence of *A. lumbridcoides* was highest among PSAC rather than SAC.

**Table 2. Estimated prevalence of soil-transmitted helminth infection by county, species, intensity, and risk group, Bomet and Narok Counties, 2021**

**<<< TABLE 2 >>>**

**<<< FIG 2 >>>**

**Fig 2. Estimated prevalence of soil-transmitted helminth infection by county, species, intensity, and risk group, Bomet and Narok Counties, 2021**

*^*^N. americanus and A. duodenale*

Co-infections

Co-infections were uncommon (Table 3). When identified, they were most frequently *A.lumbricoides* and *T.trichura,* with all risk categories in both counties recording at least one co-infection. No triple infections were detected.

**Table 3. Estimated soil-transmitted helminth coinfection prevalence by county and risk group**

**<<< TABLE 3 >>>**

# Discussion

Our findings showed moderate (10-<20%) STH infection prevalence among children in Bomet and Narok Counties. Prevalence estimates of any STH infection were above the 2% treatment threshold target according to the breaking transmission strategy [12]. Additionally, we found that MHI infection prevalence was above the WHO 2030 elimination as a public health problem target of <2% among all surveyed children [7]. These findings suggest that continued annual PC in these counties is required to make progress toward the elimination of STH morbidity as a public health problem in the country.

Compared to prior surveys, the estimated prevalences among SAC from our community-based surveys (Bomet, 16.2%, and Narok, 11.3%) were lower than those demonstrated by school-based impact surveys conducted in 2017, five years after the commencement of the National School-Based Deworming Programme. These 2017 surveys showed a reduction of STH among SAC in Bomet from 29.7% in 2012 to 18.1% in 2017 and, in Narok, from 53% in 2012 to 41% in 2017[15]. However, the prevalences are higher than those derived from the ten-year model based geostatistical impact surveys (Bomet 8.3%, and Narok 10.8%) conducted between 2021 and 2022 [25]. Despite there being an overall decline in the prevalence of STH from baseline in the country, the rate of decline in these two counties has been relatively lower, which could be explained by poor sanitation facilities, resulting in possible re-infection[26].

Parasite species

*A. lumbricoides* was the most common STH infection observed in the two counties, followed by *T. trichura,* with very few hookworm infections detected. This finding corresponds with school-based surveys conducted by the National School-based Deworming Programme, which found *A. lumbricoides* to be the most common STH during baseline, midline, and end-line surveys in the country[15]. This may be due to helminths' long lifespan in human hosts and their production of many environmentally resistant eggs, which are expelled in feces to enhance their survival and lifecycle continuation, leading to reinfection [16,27]. The survey also concurs with findings from the National School-based Deworming Programme that did not show significant reductions in the prevalence of MHI infection with *T. trichura,* where albendazole is the drug distributed. In contrast, it has been shown that a combination of ivermectin and albendazole would be the drug of choice in areas with high *T. trichura* infections[26]*.* It is also possible that reinfection occurs among the at-risk age groups, resulting in a resurgence of prevalence, as stated in a meta-analysis that showed infection after treatment occurs rapidly, particularly for *A. lumbricoides* and *T.trichura* [16]. Our results suggest that the program should continue PC administration in these two counties to reduce prevalence further.

Risk groups

Both infection prevalence and MHI infection prevalence were similar among PSAC and SAC within both counties. The consistency in high prevalence among PSACs that are routinely excluded from PC distributions highlights the fact that they should be targeted during deworming exercises as infections among this age group lead to morbidity and cognitive impairment and may even compromise the performance of PSAC on expressive language[27]. In addition, given the prevalence of similar magnitude among PSAC as seen among SAC, the Ministry may consider expanding beyond the current school-based PC platform to the broader community. This expansion beyond SAC may enable the country to accelerate progress toward eliminating STH as a public health problem in line with the Breaking Transmission Strategy and the WHO 2030 NTD Roadmap.

Limitations

The survey was designed to estimate prevalence based on county-level administrative boundaries. However, intra-county prevalence heterogeneity is likely. Hence, the program would not be able to consider the diversity of the ecological conditions that have implications on the drivers of STH transmission within the counties, contributing to the heterogeneous classification of infection. This, in turn, would affect decision-making as to where PC distribution would be most efficient and cost-effective for the program. Subsequently, with these results, PC administration would be conducted in the entire county and not in specific focal areas with a prevalence above 2%, contrary to the BTS which stipulates that the implementation unit for treatment is the ward.

The sensitivity of the Kato Katz technique in detecting hookworm eggs is reduced in low prevalence settings and with sample shipment delays between the time of collection in the field and analysis in the laboratory. To mitigate this risk, laboratories were established at the nearest health centres where teams were conducting the survey. This significantly reduced the time between sample collection and laboratory analysis. Delays in sample shipment would lead to an underestimation of the true prevalence of hookworm infections in the sampled population[30,31].

During the survey conducted in Narok County, data from one village was lost, reducing the sample size. This loss may have influenced the STH prevalence estimate in the county. However, it's anticipated that any shifts in the county-level prevalence would likely be minimal given that data from the missing village represent approximately 1/30th of the total sample size.

Conclusion

Our survey showed that STH infections remain a public health problem in Narok and Bomet counties. The elevated infection prevalence and MHI infection prevalence among all surveyed groups, including PSAC and WRA, suggests that expansion to risk groups beyond SAC during deworming exercises, as envisioned in the Breaking Transmission Strategy, may accelerate progress to controlling STH infections.

# Acknowledgments

We thank the children and parents who participated in this survey and the supervision and technical support from the Division of Vector-Borne and Neglected Tropical Diseases, Children Without Worms, and the Africa Field Epidemiology Network [AFENET]. We are grateful to the field officers who collected the data and the data team who worked tirelessly to ensure the survey was completed. We acknowledge the support from the national and county management in Bomet and Narok counties.

# References

1.	WHO. Ending the neglect to attain the Sustainable Development Goals: a road map for neglected tropical diseases 2021–2030. Geneva: World Health Organization (https://www.who.int/neglected_diseases/Revised-DraftNTD-Roadmap-23Apr2020.pdf). Word Health Organization. 2020. 196 p.

2.	Id R, Mpanya A, Inocencio R, Id SL, Id EH, Boelaert M. Community-based survey on helminth infections in Kwilu province , the Democratic Republic of the Congo , and implications for local control strategies. 2020;1–14.

3.	Brooker S. Estimating the global distribution and disease burden of intestinal nematode infections : Adding up the numbers – A review. Int J Parasitol [Internet]. 2010;40(10):1137–44. Available from: http://dx.doi.org/10.1016/j.ijpara.2010.04.004

4.	Zeleke AJ, Derso A, Bayih AG, Gilleard JS, Eshetu T. Prevalence, Infection Intensity and Associated Factors of Soil-Transmitted Helminthiasis Among School-Aged Children from Selected Districts in Northwest Ethiopia. Res Rep Trop Med. 2021;Volume 12:15–23.

5.	Jourdan PM, Lamberton PHL, Fenwick A, Addiss DG. Seminar Soil-transmitted helminth infections. Lancet [Internet]. 2017;6736(17):1–14. Available from: http://dx.doi.org/10.1016/S0140-6736(17)31930-X

6.	Bethony J, Brooker S, Albonico M, Geiger SM, Loukas A, Diemert D, et al. Soil-transmitted helminth infections : ascariasis , trichuriasis , and hookworm. 2006;367.

7.	World Health Organization (WHO). 2030 targets for soil-transmitted helminthiases control programmes. 2012;

8.	Pullan RL, Gething PW, Smith JL, Mwandawiro CS, Sturrock HJW, Gitonga CW, et al. Spatial Modelling of Soil-Transmitted Helminth Infections in Kenya : A Disease Control Planning Tool. 2011;5(2).

9.	Ministry of Health Kenya. The 2nd Kenya National Strategic Plan For control of NEGLECTED TROPICAL DISEASES 2016-2020. 2020;

10.	Ng’ang’a M, Matendechero S, Kariuki L, Omondi W, Makworo N, Owiti PO, et al. Burden of Soil Transmitted Helminthiasis in Primary school children in Migori County, Kenya. Suppl East African Med J. 2016;93(10):32–9.

11.	Ministry of Health Kenya. The 2nd Kenya National Strategic Plan For control of Neglected Tropical Diseases. 2015.

12.	Ministry of Health Kenya. THE KENYA NATIONAL BREAKING TRANSMISSION STRATEGY FOR SOIL-TRANSMITTED HELMINTHIASIS, SCHISTOSOMIASIS, LYMPHATIC FILARIASIS AND TRACHOMA. 2019;

13.	Anderson RM, Turner HC, Truscott JE, Hollingsworth TD. Should the Goal for the Treatment of Soil Transmitted Helminth ( STH ) Infections Be Changed from Morbidity Control in Children to Community-Wide Transmission Elimination ? 2020;1–8.

14.	Okoyo C, Nikolay B, Kihara J, Simiyu E, Garn J V., Freeman MC, et al. Monitoring the impact of a national school based deworming programme on soil-transmitted helminths in Kenya: The first three years, 2012 - 2014. Parasites and Vectors [Internet]. 2016;9(1):1–13. Available from: http://dx.doi.org/10.1186/s13071-016-1679-y

15.	Mwandawiro C, Okoyo C, Kihara J, Simiyu E, Kepha S, Campbell SJ, et al. Results of a national school-based deworming programme on soil-transmitted helminths infections and schistosomiasis in Kenya : 2012 – 2017. 2019;1–18.

16.	King CH, Zhou X nong, Jia T wu, Melville S. Soil-Transmitted Helminth Reinfection after Drug Treatment : A Systematic Review and Meta-Analysis. 2012;6(5).

17.	Njenga SM, Mwandawiro CS, Muniu E, Mwanje MT, Haji FM, Bockarie MJ. Adult population as potential reservoir of NTD infections in rural villages of Kwale district , Coastal Kenya : implications for preventive chemotherapy interventions policy. 2011;2–7.

18.	Njenga SM, Mutungi FM, Wamae CN, Mwanje MT, Njiru KK, Bockarie MJ. Once a year school-based deworming with praziquantel and albendazole combination may not be adequate for control of urogenital schistosomiasis and hookworm infection in Matuga District , Kwale County , Kenya. 2014;1–9.

19.	Narok County Government. Narok County Integrated Development Plan 2018-2022. 2022;1–52.

20.	County Government of Bomet. County Government of Bomet County Integrated Development Plan 2018-2022. 2018;(February 2018):518. Available from: https://cog.go.ke/cog-reports/category/106-county-integrated-development-plans-2018-2022?download=316:bomet-county-integrated-development-plan-2018-2022

21.	Kenya National Bureau of Statistics. Kenya Population and Housing Census 2019 Volume III [Internet]. Vol. 21. 2020. 1–9 p. Available from: http://journal.um-surabaya.ac.id/index.php/JKM/article/view/2203

22.	World Health Organization (WHO). Assessing the Epidemiology of Soil Transmitted Helminths during a Transmission Assessment Survey in the Global Programme for the Elimination of Lymphatic Filariasis. 2015.

23.	Katz N, Chaves A, Pellegrino J. A simple device for quantitative stool thick-smear technique in Schistosomiasis mansoni. Rev Inst Med Trop Sao Paulo. 1972;14(6):397–400.

24.	World Health Organization (WHO). Preventive chemotherapy guideline to control soil-transmitted helminth infections in at-risk population groups. 2017.

25.	Okoyo C, Minnery M, Orowe I, Owaga C, Wambugu C, Olick N, et al. Using a model-based geostatistical approach to design and analyse the prevalence of schistosomiasis in Kenya. 2023;(November):1–10.

26.	Masaku J, Okoyo C, Araka S, Musuva R, Njambi E, Njomo DW, et al. PLOS NEGLECTED TROPICAL DISEASES Understanding factors responsible for the slow decline of soil-transmitted helminthiasis following seven rounds of annual mass drug administration ( 2012 – 2018 ) among school children in endemic counties of Kenya : A mixe. 2023;1–29. Available from: http://dx.doi.org/10.1371/journal.pntd.0011310

27.	Flammer PG, Smith AL, Smith AL. Intestinal helminths as a biomolecular complex in archaeological research. 2020;

28.	Hürlimann E, Keller L, Patel C, Welsche S, Hattendorf J, Ali SM, et al. Efficacy and safety of co-administered ivermectin and albendazole in school-aged children and adults infected with Trichuris trichiura in Côte d’Ivoire, Laos, and Pemba Island, Tanzania: a double-blind, parallel-group, phase 3, randomised controlled trial. Lancet Infect Dis. 2022;22(1):123–35.

29.	Mazibuko XI, Chimbari M. The effect of schistosomiasis and soil-transmitted helminths on expressive language skills among African preschool children. BMC Infect Dis [Internet]. 2022;22(1):1–9. Available from: https://doi.org/10.1186/s12879-022-07260-2

30.	Loukas A, Hotez PJ, Diemert D, Yazdanbakhsh M, Correa-oliveira R, Croese J. Hookworm infection. 2016;

31.	Bosch F, Palmeirim MS, Ali SM, Ame SM, Hattendorf J, Keiser J, et al. Diagnosis of soil-transmitted helminths using the kato-katz technique: What is the influence of stirring, storage time and storage temperature on stool sample egg counts? PLoS Negl Trop Dis [Internet]. 2021;15(1):1–17. Available from: http://dx.doi.org/10.1371/journal.pntd.0009032
