# Supplementary material for: Prevalence and intensity of soil-transmitted helminth infections in Narok and Bomet Counties, Kenya: Evidence from program monitoring
Source: PLoS Negl Trop Dis. 2025 Dec 30;19(12):e0012415. doi: 10.1371/journal.pntd.0012415 (PMC12753061; doi:10.1371/journal.pntd.0012415)
Supplement: Attachment — Submitted filename: Response to reviewers_v1.docx [file pntd.0012415.s005.docx]

**Date: 26th April 2025**

Dear Reviewers,

We thank you and the reviewers for the thoughtful and constructive feedback provided. Below, we address each of the reviewers' comments, providing clarifications, justifications, and detailing revisions made to the manuscript. Where specific requests were not addressed, we provide our rationale, referencing the [ICSPM Reference manual](https://childrenwithoutworms.org/icspm-reference-manual/) as needed.

**Reviewe 1:**

**This work is topical and of interest in public health, in the prevention and elimination of neglected tropical diseases, and in particular intestinal parasitic infections. This work will help to provide essential, even vital, information for the management of populations in environments that continue to pay a heavy price for these parasitic affections.**

**Strength: This work is part of the implementation of the World Health Organization (WHO) 2020-2030 roadmap;**

**-From controlling morbidity to eliminating neglected tropical diseases.**

**Weakness: Low spectrum of intervention, given that this is national STH prevention**

**Response:** We thank the reviewer for recognising our study in the broader context of the WHO 2020-2030 roadmap for NTDs. With regards to the spectrum of intervention, this study focused on the two counties using the ICSPM methodology in a bid to assess reasons for ongoing transmission despite several rounds of deworming. In addition, previous studies focussed on school-going children while this study focused on three at-risk groups (PSAC, SAC and WRA).

***Methods***

***Ethical Consideration***

**Wouldn't there be a National Ethics Committee in Kenya for ethical approval?**

**Response:** Ethical approval was granted by the Amref Health Africa Ethics and Scientific Review Committee, which is accredited by the National Commission for Science, Technology and Innovation (NACOSTI). At the time of the study, this was deemed sufficient to conduct the study.

**Were children aged 2-4 able to give verbal consent? What was the benefit for participants, particularly those diagnosed as positive?**

**Response:** For children aged 2-4 years, verbal assent was provided by the parent/caregiver. While no deworming was provided immediately post-survey, participants received health education and were referred to ongoing local PC programs. This approach is consistent with the survey protocol and ethical approvals.

***Survey setting and population***

**Why did you choose particularly Narok and Bomet for this study? Is there an explanation?**

**What were the criteria used to select the composite locations?**

**Response:** We appreciate this observation and query by the reviewer regarding the selection of these counties. These counties were purposively selected due to persistent STH transmission despite multiple rounds of preventive chemotherapy. The composite locations were defined as clusters and sampling as per the [ICPSM protocol](https://childrenwithoutworms.org/icspm-reference-manual/) to ensure representativeness across the three at-risk-groups.

**The methods section is poorly structured!**

We appreciate the reviewer’s helpful feedback regarding the structure of the Methods section. In response, we have **reorganised and expanded the section** to follow a **clearer and more logical structure**, directly aligned with the **ICSPM survey methodology**.

The revised Methods section now includes the following distinct subheadings:

1. **Ethics Statement**
2. **Survey Setting and Population**
3. **Survey Design**
4. **Recruitment and Sample Collection**
5. **Laboratory Procedure**
6. **Classification of Infection Intensity**
7. **Data Collection and Management**
8. **Data Analysis**

***Statistical analysis***

**The statistical analysis section is not rich in information. No statistical tests have been applied! I suggest that you carry out comparative analyses by gender, age group (intra county) and between counties.**

**What did you do with the questionnaire data? Wasn't it possible to highlight risk factors?**

**Response:** We thank the reviewer for the comments. As per the ICSPM methodology we did not assess the risk factors in this manuscript. However, the questionnaire data were collected for future risk factor analysis, which was beyond the scope of this baseline prevalence manuscript required for programmatic decision making on PC administration.

***Results***

**The results do not follow the objectives set, but they are confused!**

**From the above, I propose the following plan:**

**i) Parasites infections;**

**ii) Intensity of infection;**

**iii) Parasites infection status, stratified by age and sex**

**iv) Co-infections probably;**

**v) Associations between parasites infections and sociodemographic factors.**

**Response:** We thank the reviewer for their suggestions regarding the results section. We have expanded the methodology section, providing further clarification. The results presented also adhere to the previously established analysis plan outlined in the ICSPM protocol.

**Discussion**

**- The discussion is descriptive rather than explanatory.**

**- The main outcome (effect of interventions on parasitic infections) is poorly documented.**

**-Page 14 line 295-297: more a recommendation than a discussion**

**The discussion explains the key results announced in the results section.**

The discussion now emphasizes that while deworming has been ongoing for nearly a decade, the prevalence of any STH infection and MHI infections remained above the WHO thresholds. We have linked this observation to potential limitations in the scope of the National School-Based Deworming Programme, which excludes PSAC and WRA. This better contextualizes the limited observed impact of the current intervention. The discussion now emphasizes that while deworming has been ongoing for nearly a decade, the prevalence of any STH infection and MHI infections remained above the WHO thresholds. We have linked this observation to potential limitations in the scope of the National School-Based Deworming Programme, which excludes PSAC and WRA. This better contextualizes the limited observed impact of the current intervention.

**The other comments were mentioned in the document**.

**Reviewer 2:**

**The objectives of the study are clearly mentioned in an own paragraph as part of the introduction. I suggest moving this part to the methods but including an additional "aim-sentence" at the end of the introduction.**

We thank the reviewer for this suggestion. An additional aim sentence has been added at the end of the Introduction, clarifying the programmatic motivation behind the

**== the authors decided for a sample size of 332 based on WHO recommendations for STH prevalence assessment in the context of LF transmission assessment surveys. However, in the present manuscript, no LF transmission assessment survey was done. Hence, I have doubts that the number can just be used for solely doing an STH prevalence assessment.**

**== The sentence describing the number 332 and the WHO recommendations does not cite the WHO recommendations but a paper by Katz**

We thank the reviewers for their insights into sample size calculations. As per the ICSPM manual, the sample size of 332 per risk group was calculated to ensure that, even with this design effect, we could classify prevalence thresholds with adequate statistical power, particularly for determining whether prevalence was below key programmatic cutoffs (e.g., <2%, <10%, <20%). This methodology has been highlighted in the ICSPM manual, the WHO manual <https://www.who.int/publications/i/item/9789241508384> and several other publications including <https://journals.plos.org/plosntds/article?id=10.1371/journal.pntd.0011605> .

This methodology ensures standardized precision for programmatic decision-making. In addition, we have inserted the correct reference to the WHO manual that describes the sample size requirement to provide the lowest point prevalence to make programmatic decisions on the need for deworming.

**== In line 153, it is not clear what defines a "district" as above the only units described are counties and sub-counties.**

All references to “district” have been corrected to reflect Kenya's county and sub-county administrative structure.

**In lines 214-216, the authors describe the calculations they conducted. I wonder why the authors decided to only provide the upper confidence interval and not the lower given that the WHO goal of 2% is rather on the lower than the upper side in this context. Furthermore, the authors present in the results different sub analyses, e.g. per species or per risk group. It would be good to mention this here, too.**

We thank the reviewers for the review and acknowlede that most publications provide both upper and lower confidence intervals. However in line with ICSPM protocol (pg. 33), we report the lowest possible point prevalence and one-sided upper confidence limits to assess whether prevalence falls below thresholds. This approach enables programmatic decisions on mass drug administration (MDA) and maintains alignment with ICSPM-based studies.

**There are no concerns about ethical or regulatory requirements being met. The authors asked for written consent of adults or legal guardians of children and verbal assent of participating children. For future studies, I would recommend, however, to include a written assent of children from the age of e.g. 8 onwards as well.**

We acknowledge the value of written assent and will consider incorporatng it in future studies for children aged 8+, although verbal assent was deemed sufficient by the ethical review board for this survey.

**Reviewer 3:**

**While the manuscript details the sampling process, it lacks a statistical justification for the sample size. The authors should explain whether the chosen sample size is powered to detect significant differences in prevalence between the risk population groups and counties. Additionally, discussing potential limitations due to non-response would add rigor.**

We thank the reviewers for their review of the statistical considerations. The sample size of 332 per risk group is validated by ICSPM and the WHO TAS manual as sufficient for classifying STH prevalence thresholds (ICSPM, pg. 12), powered to provide the lowest point prevalence at the county level. The sample size of 332 per risk group was calculated to ensure that, even with this design effect, we could classify prevalence thresholds with adequate statistical power, particularly for determining whether prevalence was below key programmatic cutoffs (e.g., <2%, <10%, <20%). The oversampling by 30% in each county was done to account for the anticipated non-response rate.

**The manuscript applies the WHO infection intensity thresholds, but it does not justify their relevance to the study setting. Given the varying endemicity levels and transmission dynamics in Narok and Bomet, a brief discussion on why these thresholds are suitable would be beneficial. This 2% threshold is a global benchmark intended to guide public health interventions. However, its applicability can vary based on local endemicity and transmission dynamics. In areas with high transmission rates, reinfection can occur rapidly after treatment, potentially leading to fluctuations in infection intensity even if the prevalence temporarily falls below 2% (see Truscott et al, 2021). Conversely, in low-endemicity settings, maintaining the 2% threshold might require sustained interventions to prevent resurgence (see Werkman et al, 2018). Therefore, while the 2% threshold serves as a useful guideline, tailoring control strategies to local conditions is essential for effective and sustainable STH management.**

We thank their reviewers for their review, ensuring that the manuscript aligns with the global STH targets. We have expanded the discussion to reflect that while the 2% threshold is a global benchmark, its application is contextualized by the need to accommodate local transmission dynamics and more importantly to inform programmatic decisions on MDA continuation, consistent with WHO guidelines and the Kenyan Breaking Transmission Strategy.

**The manuscript acknowledges the limitations of Kato-Katz in detecting hookworm eggs, particularly in low-prevalence settings, and describes a mitigation strategy by reducing sample transport time. However, even under optimal conditions, the Kato-Katz method has inherently low sensitivity for hookworm diagnosis due to rapid egg degradation and variability in egg excretion patterns. The authors should discuss whether their approach fully mitigated this limitation and consider citing studies (e.g., Barreto et al., 2017; Nikolay et al., 2014) that show how Kato-Katz underestimates hookworm prevalence. If feasible, future studies could validate findings using more sensitive diagnostic techniques, such as PCR or FLOTAC.**

The manuscript now further discusses the limitations of Kato-Katz in low-intensity settings to highlight potential underestimation of hookworm prevalence despite our mitigation strategies in reducing transport time by the use of mobile laboratories, set up in a health centre nearest to the survey site. We will consider the use of more sensitive diagnostic techniques in future studies, especially where there is evidence of low transmission.

**The ethics statement is well-documented, but given that deworming is a standard intervention, were any specific measures taken to provide treatment to infected individuals following sample collection? This should be mentioned.**

While no deworming was provided immediately post-survey, participants received health education and were referred to ongoing local PC programs. This approach is consistent with the survey protocol and ethical approvals.

**Results**

**Reviewer #2: - The analysis mainly matches the analysis plan. However, the results are more detailed that what is described in the analysis plan, i.e. the results present prevalence per e.g. species or risk group, which is not described in the methods.**

We thank the reviewer for this observation. The Methods section has been expanded to reflect the detailed analyses presented, including prevalence by species and risk group, to provide clarity on the alignment with the analysis plan.

**The figures and tables are nice and seem to have good quality. However, for one of the figures, I wonder why the authors decided to present it in black and white. A comment can be found in the attached word document.**

For production efficiency and to ensure clarity in diverse viewing formats, we chose to render the figure in black and white. This approach aligns with accessibility standards and printing considerations.

**Reviewer #3:**

**The manuscript presents prevalence estimates with upper 95% confidence limits but does not discuss how design effects were accounted for. Clarifying the method for computing confidence intervals (e.g., whether adjustments were made for clustering effects) would be helpful.**

In accordance with ICSPM standards, we assumed a design effect (DEFF) of 2.0 for our cluster-sampled survey, which is standard for similar parasitological surveys using this methodology (ICSPM manual, pg. 12).Since the ICSPM methodology produces an equal-probability sample of risk-group members within strata, all clusters in each county were selected from the same sampling frame, and analyses were kept separate for each county, no weight variables were added.

**Table 2 presents prevalence by county and risk group, but it is unclear whether these differences are statistically significant. Adding p-values or confidence intervals for comparisons would enhance interpretability.**

We thank the reviewer for these highlights. However as per the ICSPM methodology, our focus is on determining whether prevalence falls below programmatic thresholds, using the lowest point prevalence and upper, one-sided 95% confidence limits. The ICSPM framework prioritizes threshold-based decisions over inter-group statistical comparisons. Therefore, we did not calculate p-values or two-sided CIs, as our focus remained on guiding public health interventions rather than testing statistical significance across subgroups.

**Figure 2 effectively visualizes infection prevalence, but it does not highlight co-infection patterns. Given that co-infections were observed, a separate figure showing co-infection frequencies might be useful.**

Co-infection data are presented comprehensively in Table 3, showing all observed combinations of infection, stratified by county and risk group as per our initial analysis plan.

**Conclusions**

**Reviewer #2: - The discussion is supported by the data presented. However, the discussion could be made a bit broader, taking other studies and countries into account and draw conclusions not only from their own results but in relationship to other studies. I added two examples in the comments to the discussion part. The authors could write a bit more details about the future implications of their and other researcher's results.
- The limitations are clearly described
- The public health relevance is described**

We thank the reviewer for this valuable suggestion. In response, we have broadened the Discussion section to include comparative findings from other East African countries and beyond. Specifically, we have incorporated results from similar community-based STH prevalence surveys conducted in Uganda, Sierra Leone, and Bangladesh, which highlight the challenges of persistent STH transmission despite multiple rounds of mass drug administration (MDA). These comparative insights help contextualize our findings within a broader regional and global perspective.

**Reviewer #3: • The manuscript suggests expanding deworming to preschool-aged children (PreSAC) and women of reproductive age (WRA). However, it does not fully explore the operational challenges of such an expansion. A discussion on feasibility, cost implications, and alternative delivery strategies (e.g., integrating PC into maternal health services) would strengthen this recommendation**

We thank the reviewer for this important comment regarding the operational aspects of expanding preventive chemotherapy (PC) to PreSAC and WRA. We have highlighted additional evidence from Kenya and Malawi on the pilot of community based deworming, in a randomized control trial setup and the benefits attributable from a similar approach in Malawi.

**Given the persistent prevalence of moderate-to-heavy infections despite ongoing mass drug administration (MDA), it would be useful to discuss reinfection dynamics. Are there environmental or behavioural factors that could be contributing to this persistence? Linking the findings to broader discussions on water, sanitation, and hygiene (WASH) interventions could provide a more holistic interpretation.**

We thank the reviewer for highlighting the importance of reinfection dynamics and the role of environmental and behavioral factors in the persistence of STH infections. In the discussion, we take note that the persistence of moderate-to-heavy intensity infections despite ongoing MDA may be influenced by environmental conditions and WASH-related challenges, specifically, the limited access to improved sanitation and hygiene, which likely contributes to continued transmission and reinfection.

**Minor Comments:
Abstract: Consider simplifying the Background to focus more on key findings and implications. In conclusions, emphasize actionable recommendations (e.g., programmatic changes needed for STH control).**

We thank the reviewer for this suggestion. The Background section of the abstract has been simplified to more clearly highlight the study’s objectives and programmatic rationale. Additionally, the conclusions now emphasize actionable recommendations, to consider expanding treatment to other risk groups, aligned with national and WHO 2030 targets.

**Discussion: Clarify whether prevalence reductions observed in the study are statistically meaningful compared to previous estimates. Expand on the role of alternative treatment regimens (ie. incorporating ivermectin), particularly for Trichuris trichiura, which is known to be less responsive to albendazole**

We appreciate this important observation. We acknowledge that while our survey was not powered for statistical comparisons with previous surveys, moderate-to-heavy intensity infections remain above WHO thresholds, indicating limited reduction despite ongoing MDA. Additionally, we have expanded the discussion on treatment options, noting that *Trichuris trichiura* infections are less responsive to albendazole, and alternative regimens such as ivermectin-albendazole have shown improved efficacy. This supports consideration of combination therapy in areas with persistent *T. trichiura* infections**.**

**Conclusion**

We believe these revisions address the key concerns raised and enhance the clarity, rigour, and public health intervention relevance of the manuscript. Thank you for the opportunity to improve our work.

Sincerely,

Dr Paul Kibati (On behalf of all authors)
