# Supplementary material for: Prevalence and intensity of soil-transmitted helminth infections in Narok and Bomet Counties, Kenya: Evidence from program monitoring
Source: PLoS Negl Trop Dis. 2025 Dec 30;19(12):e0012415. doi: 10.1371/journal.pntd.0012415 (PMC12753061; doi:10.1371/journal.pntd.0012415)
Supplement: Attachment — Submitted filename: Response_to_Reviewers_Comments_Reviewer#1.docx [file pntd.0012415.s006.docx]

Dear Reviewer,

We thank you and for the thoughtful and constructive feedback provided. Below, we address each of the reviewers' comments, providing clarifications, justifications, and detailing revisions made to the manuscript. Where specific requests were not addressed, we provide our rationale, referencing the [ICSPM Manual](https://childrenwithoutworms.org/icspm-reference-manual/) as needed. We had combined the feedback into the reviewers feedback and apologize for the oversight in addressing specific comments

**COMMENTS**

***General comments***

**This work is topical and of interest in public health, in the prevention and elimination of neglected tropical diseases, and in particular intestinal parasitic infections. This work will help to provide essential, even vital, information for the management of populations in environments that continue to pay a heavy price for these parasitic affections.**

**Strength: This work is part of the implementation of the World Health Organization (WHO) 2020-2030 roadmap;**

**-From controlling morbidity to eliminating neglected tropical diseases.**

**Weakness: Low spectrum of intervention, given that this is national STH prevention**

**Response:** We thank the reviewer for recognising our study in the broader context of the WHO 2020-2030 roadmap for NTDs. With regards to the spectrum of intervention, this study focused on the two counties using the ICSPM methodology in a bid to assess reasons for ongoing transmission despite several rounds of deworming. In addition, previous studies focussed on school-going children while this study focused on three at-risk groups (PSAC, SAC and WRA).

***Methods***

***Ethical Consideration***

**Wouldn't there be a National Ethics Committee in Kenya for ethical approval?**

**Response:** Ethical approval was granted by the Amref Health Africa Ethics and Scientific Review Committee, which is accredited by the National Commission for Science, Technology and Innovation (NACOSTI). At the time of the study, this was deemed sufficient to conduct the study

**Reviewe#1: From now on, you have to go through the National Ethics Committee in addition to the institutional commission.**

**Were children aged 2-4 able to give verbal consent? What was the benefit for participants, particularly those diagnosed as positive?**

**Response:** For children aged 2-4 years, verbal assent was provided by the parent/caregiver. While no deworming was provided immediately post-survey, participants received health education and were referred to ongoing local PC programs. This approach is consistent with the survey protocol and ethical approvals.

**Reviewe#1: This statement comes from you “Written informed consent by parents or guardians and verbal assent by participating children** **was obtained before enrolment”.**

***Survey setting and population***

**Why did you choose particularly Narok and Bomet for this study? Is there an explanation?**

**What were the criteria used to select the composite locations?**

**Response:** We appreciate this observation and query by the reviewer regarding the selection of these counties. These counties were purposively selected due to persistent STH transmission despite multiple rounds of preventive chemotherapy. The composite locations were defined as clusters and sampling as per the [ICPSM protocol](https://childrenwithoutworms.org/icspm-reference-manual/) to ensure representativeness across the three at-risk-groups.

**Reviewe#1: Please include this information in the manuscript. Also, make the “hotspot” aspect part of the title.**

**The methods section is poorly structured!**

We appreciate the reviewer’s helpful feedback regarding the structure of the Methods section. In response, we have **reorganised and expanded the section** to follow a **clearer and more logical structure**, directly aligned with the **ICSPM survey methodology**.

The revised Methods section now includes the following distinct subheadings:

1. **Ethics Statement**
2. **Survey Setting and Population**
3. **Survey Design**
4. **Recruitment and Sample Collection**
5. **Laboratory Procedure**
6. **Classification of Infection Intensity**
7. **Data Collection and Management**
8. **Data Analysis**

**Reviewe#1: No major changes in the structure!**

***Statistical analysis***

**The statistical analysis section is not rich in information. No statistical tests have been applied! I suggest that you carry out comparative analyses by gender, age group (intra county) and between counties.**

**Reviewe#1: No response was given to this request! This is crucial!!!**

**What did you do with the questionnaire data? Wasn't it possible to highlight risk factors?**

**Response:** We thank the reviewer for the comments. As per the ICSPM methodology we did not assess the risk factors in this manuscript. However, the questionnaire data were collected for future risk factor analysis, which was beyond the scope of this baseline prevalence manuscript required for programmatic decision making on PC administration.

**Reviewe#1: We can't make any programmatic decisions without taking stock of the risk factors... that would be putting the cart before the horse! If the data is there, why not exploit it?**

***Results***

**The results do not follow the objectives set, but they are confused!**

**From the above, I propose the following plan:**

**i) Parasites infections;**

**ii) Intensity of infection;**

**iii) Parasites infection status, stratified by age and sex**

**iv) Co-infections probably;**

**v) Associations between parasites infections and sociodemographic factors.**

**Response:** We thank the reviewer for their suggestions regarding the results section. We have expanded the methodology section, providing further clarification. The results presented also adhere to the previously established analysis plan outlined in the ICSPM protocol.

**Discussion**

**- The discussion is descriptive rather than explanatory.**

**- The main outcome (effect of interventions on parasitic infections) is poorly documented.**

**-Page 14 line 295-297: more a recommendation than a discussion**

**The discussion explains the key results announced in the results section.**

The discussion now emphasizes that while deworming has been ongoing for nearly a decade, the prevalence of any STH infection and MHI infections remained above the WHO thresholds. We have linked this observation to potential limitations in the scope of the National School-Based Deworming Programme, which excludes PSAC and WRA. This better contextualizes the limited observed impact of the current intervention. The discussion now emphasizes that while deworming has been ongoing for nearly a decade, the prevalence of any STH infection and MHI infections remained above the WHO thresholds. We have linked this observation to potential limitations in the scope of the National School-Based Deworming Programme, which excludes PSAC and WRA. This better contextualizes the limited observed impact of the current intervention.

**The other comments were mentioned in the document**.
