# Supplementary material for: Prevalence and intensity of soil-transmitted helminth infections in Narok and Bomet Counties, Kenya: Evidence from program monitoring
Source: PLoS Negl Trop Dis. 2025 Dec 30;19(12):e0012415. doi: 10.1371/journal.pntd.0012415 (PMC12753061; doi:10.1371/journal.pntd.0012415)
Supplement: Attachment — Submitted filename: Response to reviewers_Aug2025.docx [file pntd.0012415.s008.docx]

**Date: 12th August 2025**

**Dear Reviewers**

We thank you and the reviewers for the thoughtful and constructive feedback provided. Below, we address each of the reviewers' comments, providing clarifications, justifications, and detailing revisions made to the manuscript.

**Reviewers' comments:**

**Reviewer's Responses to Questions**

**Key Review Criteria Required for Acceptance?

As you describe the new analyses required for acceptance, please consider the following:

Methods

-Are the objectives of the study clearly articulated with a clear testable hypothesis stated?

-Is the study design appropriate to address the stated objectives?

-Is the population clearly described and appropriate for the hypothesis being tested?

-Is the sample size sufficient to ensure adequate power to address the hypothesis being tested?

-Were correct statistical analysis used to support conclusions?

-Are there concerns about ethical or regulatory requirements being met?**

**Reviewer #1: i) Are the objectives of the study clearly articulated with a clear testable hypothesis stated?
Acceptable, Even if the link with the hypothesis is not very clear.

ii) Is the study design appropriate to address the stated objectives?
Yes, But some shortcomings in applicability.

iii) Is the population clearly described and appropriate for the hypothesis being tested?
Yes

iv) Is the sample size sufficient to ensure adequate power to address the hypothesis being tested?
approximately**

We thank the reviewers for their insights into sample size calculations. As per the ICSPM manual, the sample size of 332 per risk group was calculated to ensure that, even with this design effect, we could classify prevalence thresholds with adequate statistical power, particularly for determining whether prevalence was below key programmatic cutoffs (e.g., <2%, <10%, <20%). This methodology has been highlighted in the ICSPM manual, the WHO manual <https://www.who.int/publications/i/item/9789241508384> and several other publications including <https://journals.plos.org/plosntds/article?id=10.1371/journal.pntd.0011605> .

**v) Were correct statistical analysis used to support conclusions?
No statistical tests were actually carried out, just descriptive data. It would be necessary to compare the groups (PSAC, SAC, WRA) and according to demographic parameters (sex, age, etc.).**

We thank the reviewer for this comment. However as per ICSPM methodology and our data analysis plan, we did not assess risk factors in this manuscript. This is because the study was powered to classify prevalence thresholds per risk group, not to detect differences between subgroups

**vi) Are there concerns about ethical or regulatory requirements being met?
I would have preferred ethical approval by the National Ethics Committee. Given that this is a national issue.**

We take note of the reviewers’ comments and would like to state that ethical approval was granted by the Amref Health Africa Ethics and Scientific Review Committee, which is accredited by the National Commission for Science, Technology and Innovation (NACOSTI), the national body. At the time of the study, this was deemed sufficient to conduct the study. Future surveys will seek approval from the National Ethics Committee as per updated national requirements.

**Reviewer #2: The methods read more detailed now than last time. I am a bit confused that they are placed at the end of the manuscript, squeezed in between required information for the journal. I think this should be solved and the methods should be placed before the results. Made it a bit hard to jump back and forth between methods and results, too.**

We take note of the comments and have moved the methods section before the results The initial change was due to an editorial comment.

**Reviewer #3: The revised manuscript addresses the main concerns raised in the initial review. The study objectives are clearly stated and appropriate for the public health surveillance context. The cross-sectional design is suitable, and the target population is well defined.

Sample size justification is now aligned with ICSPM and WHO guidelines, and the statistical methods used (one-sided U95% CIs, DEFF = 2.0) are appropriate for threshold-based programmatic decisions. Ethical procedures are adequately described, including consent, health education, and referrals for treatment.

Overall, the methodology is sound, the data are well analyzed, and the revisions improve the manuscript’s clarity and relevance.**

We thank the reviewer for their comments and appreciate thei insight into improving the clarity of the manuscript.

**Results

-Does the analysis presented match the analysis plan?

-Are the results clearly and completely presented?

-Are the figures (Tables, Images) of sufficient quality for clarity?**

**Reviewer #1: i) Does the analysis presented match the analysis plan?
Acceptable with some shortcomings.

ii) Are the results clearly and completely presented?
Some aspects are missing...see section v of methods. Intra- and extra-group stratification analyses would be necessary. These results could be presented in an appendix, for example!**

We appreciate the comments made by the reviewer. However, the results presented in this paper adhere to the previously established analysis plan as outlined in the ICSPM protocol. **iii) Are the figures (Tables, Images) of sufficient quality for clarity?
Please match the places and dates in the titles of the figures and tables. Figure legends are not marked. Figure numbers do not appear! North is not shown on the map.**

Figure and table numbers have been updated.

**Reviewer #2: The results read fine and are presented in tables and figures. Here some comments on the results:
- I think it would be nice to include a flowchart to show the recruitment process in more detail and include i) in how many households was nobody home or no adult/head of household, ii) in how many households, individuals did not want to consent, iii) how many individuals consented but did not provide a stool sample, iv) how many individuals were excluded because they did not fulfil the eligibility criteria such as staying in the household the night before and after.
- I think a logistic regression would have been a nice add-on to this publication. I think it is not a must but would give the presentation more substance.
- The caption of Figure 2 should include the abbreviation used for the legend of the Figure. I am aware that the heading in the figure itself includes it but for completeness I would include it in the caption or even consider to exchange the HMI abbreviation for the full wording in the figure.**

We take note of the comment from the reviewer and have updated the caption of Figure 2 to match that used in the legend.

**Reviewer #3: The analysis presented is consistent with the ICSPM methodology and aligns with the stated objectives and analysis plan. The results are clearly and comprehensively presented, including stratification by species, risk group, and infection intensity. Figures and tables are of good quality and effectively support the findings. The use of Table 3 to present co-infection patterns is appropriate and sufficiently detailed.**

We appreciate the comments made by the reviwer to improve the readability of the manuscript.

**Conclusions

-Are the conclusions supported by the data presented?

-Are the limitations of analysis clearly described?

-Do the authors discuss how these data can be helpful to advance our understanding of the topic under study?

-Is public health relevance addressed?**

**Reviewer #1: i) Are the conclusions supported by the data presented?
Yes

ii) Are the limitations of analysis clearly described?
Not really!

iii) Do the authors discuss how these data can be helpful to advance our understanding of the topic under study?
Yes, but a few more sentences would be necessary!

v) Is public health relevance addressed?
You could put it this way. Authors should highlight the importance of the study to the public and the scientific community.**

**Reviewer #2: The discussion needs a bit more discussion.
Two examples:
1. The authors write: "Despite there being an overall decline in the prevalence of STH from baseline in the country, the rate of decline in these two counties has been relatively lower, which could be explained by poor sanitation facilities, resulting in possible re-infection[26]." -- So the prevalence declined but not as much and you give poor sanitation facilities as the reason. I would suggest to discuss how these could be improved. Were they already improved a little bit, therefore the decline? Where does the decline come from if its not the sanitation facilities? What would the counties need to improve them, of who is this the responsibility? Also, how did other countries manage to bring the prevalence down? Was it also because of WASH facilities or are there any other ways to reduce prevalence?**

We acknowledge the reviewers request to flesh out the discussion regarding the overall decline in prevalence. We have included additional information from geostatistical modelling that highlights some of the reasons that concur with this finding. **2. The authors write: "In contrast, it has been shown that a combination of ivermectin and albendazole would be the drug of choice in areas with high T. trichura infections[26]. Additionally, it has been shown that there is heterogeneity of STH transmission, which would necessitate a different approach in areas found to have high T. trichuris infections.(41). It is also possible that reinfection occurs among the at-risk age groups, resulting in a resurgence of prevalence, as stated in a meta-analysis that showed infection after treatment occurs rapidly, particularly for A. lumbricoides and T.trichura [16]." -- This paragraph reads incomplete to me. You write about a drug combination but you do not discuss how this might impact the prevalence in your setting. You write about heterogeneity and a different approach but you do not mention the approach or what this would mean for your counties or elsewhere. And you write about reinfections but you do not discuss how they could be prevented.**

We thank the reviewer for this comment. We have added information on the impact of deworming programs on the prevalence of infection as well as mitigation measures on how to prevent reinfection using WASH and behaviour change strategies.

**Reviewer #3: The conclusions are well supported by the data presented. The manuscript clearly describes limitations, including diagnostic sensitivity and geographic heterogeneity. The discussion effectively contextualizes findings within broader regional experiences and outlines implications for expanding preventive chemotherapy. The public health relevance is well articulated, particularly regarding policy decisions to reach underserved risk groups and sustain progress toward STH elimination targets.**

**Editorial and Data Presentation Modifications?

Use this section for editorial suggestions as well as relatively minor modifications of existing data that would enhance clarity. If the only modifications needed are minor and/or editorial, you may wish to recommend “Minor Revision” or “Accept”.**

**Reviewer #1: Authors should review the order of the writing plan! The "Methods" section appears after the acknowledgments... I don't know if this is a requirement of the editorial line!**

We thank the reviewer for this observation. The editorial team had suggested the movement of the methods section to the end which has now been reversed for easier reading.

**Reviewer #2: lines 76-79: Reference number 3 is from 2010 and therefore rather old. And reference number 4 is referring to a study solely conducted in Ethiopia. It does not seem to be a good fit for the sentence here. What about the WHO weekly epidemiological report from 2024?**

We thank the reviewer for this important information. We have included the population requiring PC and those treated within the WHO African region from the WHO weekly epidemiological record in the manuscript in line 90-92.

**line 88 and many others: There is no consistency in presenting the references. Sometimes it is a squared bracket, somethings its round brackets and in this case here its both. I would recommend to unify them and then check if they are all still correct or if because of the different referencing methods, there has been a mix of references.**

We thank the reviewer for this observation, We have updated the referencing style to be consistent with the journal requirements.

**line 119: Are there multiple Bomet Counties?**

There is only one Bomet county. We use the expression Narok and Bomet counties to signify more than one area.

**line 149: Exchange to lumbricoides**

Thank you for the keen observation, this has been corrected.

**line 319: Here, your inclusion criteria is verbal assent or consent. But further up in your ethics, you write about written consent. Which one is true?**

We thank the reviewer for this observation. We have updated the manuscript to read verbal informed consent by the parents or guardians.

**Reviewer #3: The revised manuscript is well written and clearly presented. Figures and tables are appropriate and support the findings effectively. Minor editorial suggestions include ensuring clarity in a few expanded discussion points—particularly around the feasibility and cost implications of expanding PC delivery to preschool-aged children and women of reproductive age. While the authors cite relevant pilot experiences, a more explicit discussion of implementation challenges (e.g., health system integration, community delivery platforms) would further strengthen the operational relevance of the recommendations.
These are minor suggestions that do not affect the overall conclusions.**

**Summary and General Comments

Use this section to provide overall comments, discuss strengths/weaknesses of the study, novelty, significance, general execution and scholarship. You may also include additional comments for the author, including concerns about dual publication, research ethics, or publication ethics. If requesting major revision, please articulate the new experiments that are needed.**

**Reviewer #1: Methods
Ethical Consideration
Wouldn't there be a National Ethics Committee in Kenya for ethical approval?
Response: Ethical approval was granted by the Amref Health Africa Ethics and Scientific Review Committee, which is accredited by the National Commission for Science, Technology and Innovation (NACOSTI). At the time of the study, this was deemed sufficient to conduct the study
Reviewe#1: From now on, you have to go through the National Ethics Committee in addition to the institutional commission.**

We thank the reviewer for their comment. Ethical approval was granted by the Amref Health Africa Ethics and Scientific Review Committee, which is accredited by the National Commission for Science, Technology and Innovation (NACOSTI), the national body. At the time of the study, this was deemed sufficient to conduct the study. Future surveys will seek approval from the National Ethics Committee as per updated national requirements.
 **Were children aged 2-4 able to give verbal consent? What was the benefit for participants, particularly those diagnosed as positive?**

**Response: For children aged 2-4 years, verbal assent was provided by the parent/caregiver. While no deworming was provided immediately post-survey, participants received health education and were referred to ongoing local PC programs. This approach is consistent with the survey protocol and ethical approvals.
Reviewe#1: This statement comes from you “Written informed consent by parents or guardians and verbal assent by participating children was obtained before enrolment”.**

We thank the reviewer for their comments. For children aged 2-4 years, verbal assent was provided by the parent/caregiver and updated explicitly in the manuscript. While no deworming was provided immediately post-survey, participants received health education and were referred to ongoing local PC programs. This approach is consistent with the survey protocol and ethical approvals.

**Survey setting and population
Why did you choose particularly Narok and Bomet for this study? Is there an explanation?
What were the criteria used to select the composite locations?
Response: We appreciate this observation and query by the reviewer regarding the selection of these counties. These counties were purposively selected due to persistent STH transmission despite multiple rounds of preventive chemotherapy. The composite locations were defined as clusters and sampling as per the ICPSM protocol to ensure representativeness across the three at-risk-groups.
Reviewe#1: Please include this information in the manuscript. Also, make the “hotspot” aspect part of the title.**

We thank the reviewer for their comments. As earlier indicated, These counties were purposively selected due to persistent STH transmission despite multiple rounds of preventive chemotherapy. We have updated the criteria in the manuscript as suggested. However, we have not included these areas as hotspots as it doesn’t conform to the WHO definition of hotspots as highlighted in the [WHO M&E framework for assessing schistosomiasis and soil transmitted helminthiases control programmes](https://iris.who.int/bitstream/handle/10665/379352/9789240099364-eng.pdf?sequence=1&isAllowed=y)

**The methods section is poorly structured!
We appreciate the reviewer’s helpful feedback regarding the structure of the Methods section. In response, we have reorganised and expanded the section to follow a clearer and more logical structure, directly aligned with the ICSPM survey methodology.
The revised Methods section now includes the following distinct subheadings:
1. Ethics Statement
2. Survey Setting and Population
3. Survey Design
4. Recruitment and Sample Collection
5. Laboratory Procedure
6. Classification of Infection Intensity
7. Data Collection and Management
8. Data Analysis
Reviewe#1: No major changes in the structure!**

We maintained the methods section as is as this aligns with the [STROBE checklists](https://www.strobe-statement.org/checklists/) for cross sectional analysis and our initial data analysis plan.

**Statistical analysis
The statistical analysis section is not rich in information. No statistical tests have been applied! I suggest that you carry out comparative analyses by gender, age group (intra county) and between counties.
Reviewe#1: No response was given to this request! This is crucial!!!
What did you do with the questionnaire data? Wasn't it possible to highlight risk factors?
Response: We thank the reviewer for the comments. As per the ICSPM methodology we did not assess the risk factors in this manuscript. However, the questionnaire data were collected for future risk factor analysis, which was beyond the scope of this baseline prevalence manuscript required for programmatic decision making on PC administration.**

We thank the reviewer for this comment. However as initially indicated, As per ICSPM methodology and our data analysis plan, we did not assess risk factors in this manuscript. This is because the study was powered to classify prevalence thresholds per risk group, not to detect differences between subgroups. **Reviewe#1: We can't make any programmatic decisions without taking stock of the risk factors... that would be putting the cart before the horse! If the data is there, why not exploit it?**

We thank the reviewer for making this observation. However, programmatic decisions to conduct MDAs, which is the focus of this paper, are based on prevalence thresholds and not the risk factors associated with infection. The risk factors collected are used for other routine decisions which were not the intended focus of this paper.

**Reviewer #2: I think the authors have targeted an important topic, and its important to know the local prevalences of Counties for STH. However, I think the discussion needs a bit more discussion, including what the results mean for the implementation of interventions for the area and how they could be implemented. What is needed to reduce the prevalence in the area and how can this be solved? What does the WHO say and how did other NTD programmes or studies successfully reduced prevalence? What are the implications of your study?**

We thank the reviewer for this comment and we have included other measures required not only to reduce the prevalence but also sustain the gains made through deworming programs.

**Reviewer #3: Major comments
• Justification of Sample Size and Design Effect: The authors have now provided sufficient justification for the sample size based on ICSPM (Integrated Community-based Survey for Program Monitoring) and WHO TAS guidelines, explaining that the sample allows for classifying prevalence thresholds with appropriate power. They also clarified the assumed design effect (DEFF = 2.0), consistent with ICSPM methodology. This addresses previous concerns.
• Applicability of WHO’s 2% Threshold: The revised manuscript includes a more nuanced discussion about the relevance of the 2% MHI threshold, referencing literature (e.g., Truscott et al., 2021; Werkman et al., 2018) to explain how transmission dynamics may affect interpretation. This addition strengthens the discussion and aligns the study with programmatic and contextual considerations.
• Kato-Katz Limitations and Diagnostic Sensitivity: The manuscript now adequately acknowledges that even with mitigation measures (use of local/mobile laboratories), Kato-Katz likely underestimates hookworm prevalence in low-endemicity settings. The proposed references were appropriately included. Future use of more sensitive diagnostics (e.g., PCR) is acknowledged.
• Operational Feasibility of Expanding PC to PreSAC and WRA: The authors have expanded the discussion by referencing pilot experiences in Kenya and Malawi.
• Consideration of Reinfection and WASH: The revised discussion addresses how poor sanitation and limited access to WASH contribute to persistent transmission and reinfection. This strengthens the public health interpretation and links prevalence findings to broader structural issues.
• Interpretation of Prevalence Changes Over Time: While the manuscript notes that the survey was not powered to detect statistically significant changes from earlier surveys, it now clarifies that MHI prevalence remains above elimination thresholds, justifying the need for continued MDA.
• Alternative Treatments for Trichuris trichiura: The revised manuscript includes a brief but important mention of ivermectin-albendazole combination therapy, citing recent studies. This addresses concerns about the limited efficacy of albendazole for T. trichiura.

Minor Comments
• The authors clarified that one-sided upper confidence intervals were used to assess if prevalence is below thresholds, following ICSPM protocol. While two-sided intervals or p-values might enhance comparative interpretation, the current approach aligns with the survey’s programmatic objectives.
• The revised ethics section now specifies that participants received health education and referrals to existing programs. This addresses the concern regarding post-survey follow-up in the absence of on-site treatment.
• The abstract has been revised to emphasize the main findings and recommendations. The conclusions are now more actionable, suggesting programmatic expansion of PC to underserved groups.
• Although a separate figure for co-infections was not included, the data are clearly presented in Table 3. This is acceptable and consistent with the structure of the manuscript.**

We thank the reviewer for the important insights in improving the clarity and potential impact of the findings to the scientific community.
