# Supplementary material for: Prevalence and intensity of soil-transmitted helminth infections in Narok and Bomet Counties, Kenya: Evidence from program monitoring
Source: PLoS Negl Trop Dis. 2025 Dec 30;19(12):e0012415. doi: 10.1371/journal.pntd.0012415 (PMC12753061; doi:10.1371/journal.pntd.0012415)
Supplement: Attachment — Submitted filename: PNTD-D-24-01087_R2_GC_09.09.2025.docx [file pntd.0012415.s009.docx]

**PLOS Neglected Tropical Diseases**

**Prevalence and intensity of soil-transmitted helminth infections in Narok and Bomet Counties, Kenya: Evidence from program monitoring**

--Manuscript Draft--

| **Manuscript Number:** | PNTD-D-24-01087R2 |
| --- | --- |
| **Full Title:** | Prevalence and intensity of soil-transmitted helminth infections in Narok and Bomet Counties, Kenya: Evidence from program monitoring |
| **Short Title:** | Prevalence and intensity of soil-transmitted helminth infections in Kenya |
| **Article Type:** | Research Article |
| **Keywords:** | Helminthiasis, Public Health, Prevalence, Morbidity, kenya |
| **Abstract:** | Background  The backbone of Kenya's soil-transmitted helminthiasis control program is the periodic distribution of anti-helminthic Preventive Chemotherapy (PC), primarily through the National School-Based Deworming Programme, which has targeted school-age children (SAC, 5–14 years) living in areas at high risk for soil-transmitted helminth (STH) infections since 2012. After nearly a decade of deworming, recent evidence suggest that transmission may still occur, especially among other at-risk groups not routinely treated, such as preschool aged children (PSAC, 2-4 years) and women of reproductive age (WRA 15-49 years). The study sought to generate evidence for program monitoring and continued PC administration by estimating the prevalence and intensity of STH infection in Narok and Bomet counties among PSAC, SAC and WRA. Methods  During August and December of 2021, we conducted cross sectional population-based household surveys in Narok and Bomet counties, respectively, using a multi-stage, cluster random sampling among resident PSAC, SAC, and WRA. Individual and household questionnaires were administered using an electronic mobile platform. Stool samples were collected and tested for roundworms (Ascaris lumbricoides), hookworms (Necator americanus, Ancylostoma duodenale), and whipworms (Trichuris trichura) using duplicate thick-smear Kato-Katz microscopy.  Results  Stool samples were provided by 1,062 PSAC, 1,922 SAC, and 364 WRA. Results indicated that the prevalence of any STH infection was highest among SAC (16.2% [upper 95% design-corrected confidence interval [U95% CI]: 23.1%]), followed by PSAC in Bomet county (15.8% [U95% CI: 21.7%]). In Narok county, STH prevalence was highest among PSAC (12.8% [U95% CI: 18.2%]) followed by SAC (11.3% [U95% CI: 16.2%]). Moderate-to-high intensity infection prevalence among PSAC and SAC exceeded the morbidity elimination threshold of 2% in both counties. A. lumbricoides and T. trichura were most identified; few hookworm infections were detected.  Conclusions  STH infections remain a public health problem in Narok and Bomet counties. There may be a need to expand PC distribution to include risk groups beyond SAC during deworming exercises, as envisioned in the Breaking Transmission Strategy, to hasten progress toward the achievement of STH as a public health problem. |
| **Additional Information:** |  |
| **Question** | **Response** |
| **Government Employee**  Are you or any of the contributing authors an employee of the United States government?  Manuscripts authored by one or more US | No - No authors are employees of the U.S. government. |

| Government employees are not copyrighted, but are licensed under a [CC0](https://creativecommons.org/about/cc0) [Public Domain Dedication](https://creativecommons.org/about/cc0), which allows unlimited distribution and reuse of the article for any lawful purpose. This is a legal requirement for US Government employees.  This will be typeset if the manuscript is accepted for publication. |  |
| --- | --- |
| **Financial Disclosure**  Enter a financial disclosure statement that describes the sources of funding for the work included in this submission. Review the [submission guidelines](http://journals.plos.org/plosntds/s/submission-guidelines#loc-financial-disclosure-statement) for detailed requirements. View published research articles from [*PLOS Neglected Tropical*](https://journals.plos.org/plosntds/) [*Diseases*](https://journals.plos.org/plosntds/) for specific examples.  This statement is required for submission and **will appear in the published article** if the submission is accepted. Please make sure it is accurate. | The author(s) received no specific funding for this work. |
| **Funded studies**  Enter a statement with the following details:   - Initials of the authors who received each award - Grant numbers awarded to each author - The full name of each funder - URL of each funder website - Did the sponsors or funders play any role in the study design, data collection and analysis, decision to publish, or preparation of the manuscript? |  |
| Did you receive funding for this work? |  |
| **Competing Interests**  On behalf of all authors, disclose any competing interests that could be perceived to bias this work.  This statement will be typeset if the | I have read the journal’s policy and the authors of the manuscript have the following competing interests: KMS receives a salary from The Task Force for Global Health, an organization that receives funding from GSK and Johnson & Johnson, the manufacturers of albendazole and mebendazole, respectively. |

| manuscript is accepted for publication.  Review the instructions link below and PLOS NTDs’ [competing interests](https://journals.plos.org/plosntds/s/competing-interests#loc-what-represents-a-competing-interest) policy to determine what information must be disclosed at submission. |  |
| --- | --- |
| **Data Availability**  Provide a **Data Availability Statement** in the box below. This statement should detail where the data used in this submission can be accessed. This statement will be typeset if the manuscript is accepted for publication.  Before publication, authors are required to make all data underlying their findings fully available, without restriction. Review our [PLOS Data Policy](https://journals.plos.org/plosntds/s/data-availability) page for detailed information on this policy. Instructions for writing your Data Availability statement can be accessed via the Instructions link below. | The data used for the analyses in this paper are owned by the government of Kenya, and are subject to provisions of the Data Protection Act https://kenyalaw.org/kl/fileadmin/pdfdownloads/LegalNotices/2021/LN263_2021.pdf . Researchers are welcome to request access to de-identified datasets from the Division of Vector-Borne and Neglected Tropical Diseases at the Ministry of Health [(headdvbntd@gmail.com).](mailto:(headdvbntd@gmail.com) |

<https://www2.cloud.editorialmanager.com/pntd/download.aspx?id=1474875&guid=6cf5d246-fd33-40b2-8c6c-109d8f34b3cb&scheme=1>Manuscript	[Click here to](https://www2.cloud.editorialmanager.com/pntd/download.aspx?id=1474875&guid=6cf5d246-fd33-40b2-8c6c-109d8f34b3cb&scheme=1)

[access/download;Manuscript;manuscript_v4_14082025.docx](https://www2.cloud.editorialmanager.com/pntd/download.aspx?id=1474875&guid=6cf5d246-fd33-40b2-8c6c-109d8f34b3cb&scheme=1)

1

2 **Prevalence and intensity of soil-transmitted helminth infections in Narok and Bomet**
3 **Counties, Kenya: Evidence from program monitoring**

4

5 Paul Kibati^1,2*^, Florence Wakesho^2^, Stephen Mwatha^1,2^, Denver Mariga^1,2^, Josephine Ihahi^1,2^,
6 Dickson Kioko^2^, Joseph Otieno Oloo^2^, Elvis Oyugi^1^, Sammy M. Njenga^3^, Ben Masiira^4^,
7 Maurice Owiny^1,4^, Cara Tupps^5^, Mariana Stephens^5^, Paul Emerson^5^, Wyckliff Omondi^2^,
8 Rubina Imtiaz^5^, Sultani Matendechero^6^, Sanjaya Dhakal^5^ and Kristin M. Sullivan^5^

9

10 ^1^*Field Epidemiology and Laboratory Training Program*, *Ministry of Health, Nairobi, Kenya*
11 ^2^*Division of Vector-Borne and Neglected Tropical Diseases, Ministry of Health, Nairobi,*
12 *Kenya*
13 *^3^Kenya Medical Research Institute, Nairobi, Kenya*
14 *^4^Africa Field Epidemiology Network, Kampala, Uganda*
15 *^5^Children Without Worms, The Task Force for Global Health, Decatur, Georgia, United*
16 *States of America*
17 *^6^National Public Health Institute, Nairobi, Kenya*

18

19

20

21

22 *[pgkibati@gmail.com](mailto:pgkibati@gmail.com)

# 23 Abstract

24 **Background**
25 The backbone of Kenya's soil-transmitted helminthiasis control program is the periodic
26 distribution of anti-helminthic preventive chemotherapy (PC). PC distribution has been
27 conducted since 2012 through the National School-Based Deworming Programme, which
28 deworms school-age children (SAC, 5–14 years) living in areas at high risk for soil-
29 transmitted helminth (STH) infections. After nearly a decade of deworming, the Ministry of
30 Health sought to generate evidence for program monitoring by estimating the prevalence and
31 intensity of STH infection in Narok and Bomet counties among at-risk groups: preschool-
32 aged children (PSAC, 2–4 years), SAC, and women of reproductive age (WRA, 15–49 years),
33 of which only SAC are covered by the deworming program.

# 34 Methods

35 During August and December of 2021, we conducted cross-sectional, population-based
36 household surveys in Narok and Bomet counties, using multi-stage, cluster random sampling
37 among resident PSAC, SAC, and WRA. Individual and household questionnaires were
38 administered using an electronic mobile platform. Stool samples were collected and tested for
39 roundworms (*Ascaris lumbricoides*), hookworms (*Necator americanus, Ancylostoma*
40 *duodenale*), and whipworms (*Trichuris trichura*) using the Kato-Katz method.

# 41 Results

42 Stool samples were provided by 1,062 PSAC, 1,922 SAC, and 364 WRA. Results indicated
43 that the prevalence of any STH infection in Bomet county was similar among SAC (16.2%
44 [upper 95% design-corrected confidence interval [U95% CI]: 23.1%]) and PSAC (15.8%
45 [U95% CI: 21.7%]). In Narok county, STH prevalence was marginally higher among PSAC
46 (12.8% [U95% CI: 18.2%]) compared to SAC (11.3% [U95% CI: 16.2%]). Moderate-to-high
47 intensity infection prevalence among PSAC and SAC exceeded the morbidity elimination
48 threshold of 2% in both counties. *A. lumbricoides* and *T. trichura* were most identified; few
49 hookworm infections were detected.

# 50 Conclusions

51 STH infections remain a public health problem in Narok and Bomet counties. There may be a
52 need to expand PC distribution to include risk groups beyond SAC during deworming
53 exercises, as envisioned in the Breaking Transmission Strategy, to hasten progress toward the
54 achievement of STH as a public health problem.

55

# 56 Author summary

57 Soil-transmitted helminth (STH) infections, including roundworms (*Ascaris lumbricoides*),
58 hookworms (*Necator americanus, Ancylostoma duodenale*), and whipworms (*Trichuris*
59 *trichura*), have been shown to affect children's nutrition and cognitive development.
60 Monitoring the prevalence of STH infections among at-risk populations remains a key step in
61 determining the type and frequency of deworming medication to administer. Globally,
62 treatment guidelines recommend treating children and women of reproductive age (WRA), as
63 they are at the highest risk of morbidity from infection. In Kenya, preventive chemotherapy
64 (PC) administration has primarily targeted school-aged children (SAC) using a school-based
65 delivery platform. However, program monitoring surveys demonstrated that the prevalence
66 and intensity of STH infections among children continues to exceed the country’s elimination
67 targets. Our results showed that in Narok and Bomet counties, the prevalence of any STH
68 infection was moderate (10–<20%) among children in both counties and low (2–<10%)
69 among WRA. This suggests the need to consider expanding PC administration to a
70 community-based model to make significant strides toward eliminating soil transmitted
71 helminths as a public health problem.

72

# 73 Introduction

74 Soil-transmitted helminth (STH) infections caused by roundworms (*Ascaris lumbricoides*),
75 hookworms (*Necator americanus*, *Ancylostoma duodenale*), and whipworms (*Trichuris*
76 *trichura*) are a public health problem globally, predominantly in the poorest regions of the
77 world [1,2]. The World Health Organization (WHO) classifies soil-transmitted helminthiasis
78 among the neglected tropical diseases (NTDs) and it is estimated that STH affects more than
79 1.5 billion people globally[3]. In 2021, it was estimated that there were more than 640 million
80 STH cases, resulting in more than 3400 deaths and 1.38 million disability-adjusted life
81 years[4].
82 STHs cause human infection through the ingestion of eggs from contaminated food
83 and soil or by active skin penetration in the case of hookworms [5]. STH infections are known
84 to cause both acute and chronic disease conditions such as anaemia, malnutrition, abdominal
85 pain and discomfort, diarrhoea, as well as impaired physical and cognitive development [5,6].
86 WHO recommends controlling morbidity caused by STH infections through preventive
87 chemotherapy (PC) with the anthelmintic drugs albendazole or mebendazole [7].
88 In Kenya, STH infections are widely distributed, occurring mainly in western and
89 southeastern coastal regions, as well as other localized areas [5]. According to Kenya’s 2016-
90 2020 NTD strategic plan [9], it is estimated that 10 million people nationwide are affected by
91 STH, with children accounting for 50%. Another 16.6 million people are deemed to be at risk
92 of contracting STH infections [9–11]. The Ministry of Health, through the Division of
93 Vector-Borne and Neglected Tropical Diseases, launched the Breaking Transmission Strategy
94 in 2019, which aimed to eliminate morbidity due to STH infections [19]. This strategy seeks
95 to integrate interventions by focusing on increasing coverage of mass drug administration
96 (MDA), establishing sustainable implementation of necessary NTD-related water, sanitation,
97 and hygiene interventions, and implementing behaviour change communication interventions
98 that are necessary to move from control to elimination of STH infections as a public health
99 problem [13].
100 The launch of the Breaking Transmission Strategy was necessitated by the fact that
101 since 2012, PC has been provided by the National School-Based Deworming Programme
102 targeting school-aged children (SAC, 5-14 years) in various parts of the country [14]. Within
103 the first three years of implementation, the program demonstrated a reduction of moderate-to-
104 heavy intensity (MHI) infection [14]. However, it was noted that STH reinfections remained
105 common following treatment, suggesting the need for expanded interventions [15,16] and
106 that, in areas of high prevalence, adults and children not in school acted as reservoirs of STH,
107 thereby impeding the effectiveness of STH control programs through school-based
108 interventions only [17,18].
109 Given this concern, in 2021, the Ministry of Health Kenya, through the Division of
110 Vector-Borne and Neglected Tropical Diseases, sought to better understand the community-
111 wide prevalence of infection. They selected two counties, Narok and Bomet, where there had
112 been minimal reduction in STH prevalence among SAC, despite ongoing school-based
113 deworming efforts spanning over five years [15]. This survey aimed to provide programmatic
114 evidence supporting community-wide deworming by determining the point prevalence and
115 intensity of STH infections among PSAC, SAC, and WRA in Narok and Bomet Counties
116 [21–23].

# 117 Objective

118 The objective of these community-based, cross-sectional surveys was to determine the
119 prevalence and intensity of STH infections among preschool-aged children (PSAC, ages 2–4
120 years), SAC (ages 5–14 years), and women of reproductive age (WRA, 15–49 years) in
121 Narok County and Bomet Counties.

# 122 Methods

123 **Ethics statement**
124 Ethical approval for the surveys was granted by the Ethics and Scientific Review Committee
125 of AMREF Health Africa in Kenya (P 705 – 2019). Permission to collect data was obtained
126 from the respective county/sub-county health offices. Participants and guardians were
127 provided with information about the purpose of the survey and what to expect. Written
128 informed consent by parents or guardians and verbal assent by participating children was
129 obtained before enrolment. While this survey did not provide immediate treatment post-
130 sample collection, all individuals and guardians of participating children received
131 comprehensive health education regarding STH transmission, prevention and treatment
132 availability through local health services.

# 133 Survey setting and population

134 The surveys were conducted in Narok and Bomet counties (Fig. 1) in August and December
135 2021, respectively. These counties were purposefully selected due to their varying ecological
136 and epidemiological contexts, historical PC administration through the National School
137 Based Deworming Program from 2012, and ongoing concerns about persistent transmission
138 of STH.
139 Narok County covers an area of 17,933 km^2^ with a population of roughly 1.2 million. The
140 county is administratively divided into six sub-counties and 30 wards, each serving as smaller
141 administrative sub-units of a sub-county[19].
142 Bomet County has an area of 2,037 km^2^ with a projected population of roughly 900,000. The
143 county is divided into five sub-counties and 25 wards[20].

144

145

146	**<<<Figure 1>>>>**

147

148

149

150

151

152

**Figure 1. Map of Kenya showing the locations of Bomet and Narok counties.**

*County and national boundaries were derived from openly licensed shapefiles provided by the United Nations Office for the Coordination of Humanitarian Affairs (OCHA) via the Humanitarian Data Exchange (HDX) platform* [*(https://data.humdata.org/dataset/cod-ab-ken)*](https://data.humdata.org/dataset/cod-ab-ken)*.*

*Map created using R version 4.3.2, with ggplot2 on 28APR2022*

# Survey design

153 We conducted a community-based, cross-sectional survey using a multi-stage cluster
154 sampling approach following the Integrated Community-based Survey for Program
155 Monitoring methodology. Similar sampling designs have been implemented in similar
156 epidemiological studies in Uganda, Sierra Leone and Bangladesh [24–26]. Full
157 methodological details can be found in the online reference manual
158 (<https://childrenwithoutworms.org/icspm-reference-manual/>), with key details provided
159 below.
160 The population-based, household surveys were conducted using a multi-stage, cluster
161 random sampling technique, producing equal selection probabilities among resident PSAC,
162 SAC, and WRA in the respective counties. Within each county, population and housing data
163 from the Kenya National Bureau of Statistics, supplemented by county administrators, were
164 used to identify the villages, which served as clusters [21]. Thirty clusters were randomly
165 selected from each county in the first stage using population proportional to estimated size.
166 The team selected clusters using the Survey Sample Builder developed by the Neglected
167 Tropical Disease Support Centre at the Task Force for Global Health
168 (<https://childrenwithoutworms.org/icspm-survey-sample-builder-tool/>).
169 The sample size for this survey was based on the transmission assessment survey
170 methodology published by WHO, which recommends at least 332 participants for each at-
171 risk group in a cluster survey[30,31]. Sample size calculation targeted the lowest precision
172 adequate for assessing whether the prevalence of any STH infection had fallen below WHO
173 thresholds for public health intervention [28,29].
174 To ensure the sample size was reached, 475 participants were selected for inclusion
175 for each risk group in each county to accommodate an approximate non-response of 30%.
176 Non-response was anticipated to cover both participants who did not consent to be surveyed
177 and those who did not provide stool samples. Households were systematically selected using
178 a pre-determined sampling interval, and the household selection form aided field teams in
179 knowing which risk group was to be sampled from the household.

# 180 Recruitment and sample collection

181 Household members of selected households were included if they: a) were a member of the
182 selected risk groups of interest (PSAC, SAC, and WRA), b) slept at the household the night
183 before the interview, c) planned to stay at the household the night of the interview, d) were
184 able to provide a single stool sample within 24 hours of the interview, and e) were able and
185 willing to provide verbal consent or assent.
186 On the survey day, field officers visited the selected households, and explained the
187 purpose of the survey and ensured consent/assent was obtained. The field teams were
188 comprised of a national-level supervisor from the Ministry of Health, a county-level
189 supervisor, a community health assistant (CHA) from the area, and a community health
190 volunteer (CHV) from the village being visited. Each participant was assigned a unique
191 identification number, which was used to track the collected sample and link it to the
192 individual information collected during the household visit. Participants were interviewed by
193 trained data collectors using a pre-tested, structured questionnaire. Socio-demographic
194 information on the household was provided by the household head/designate, and everyone
195 provided their individual-level information. Information for young children was provided by
196 the household head/designate.
197 The participants were given a labelled wide-mouthed plastic container for stool
198 collection, a piece of plain paper, a piece of applicator sticks, and other sanitary necessities
199 such as tissue paper. The plastic containers were labelled with the participants' unique
200 identification numbers and their name, age, and sex to ensure a mix-up of containers did not
201 occur within the household. The survey information was given to the head of household or
202 his designate if the household head was absent. If the household head was not conversant in
203 English, the community health assistant explained the instructions in the local language. The
204 field team explained safe stool collection to the participant and asked them to wash their
205 hands with soap and water after providing samples. The fresh stool samples were collected
206 the morning after the household visit and interview.

# 207 Laboratory procedure

208 Stool samples were transported to the laboratory using cooler boxes within three hours of
209 collection. To address the recognized sensitivity limitations of the Kato-Kats technique,
210 particularly for hookworm detection due to rapid egg degradation in low transmission
211 settings, we established mobile laboratories at the nearest health centre to minimize specimen
212 transport time [30]. Stool samples were then prepared using a 41.7 mg Kato-Katz template,
213 with duplicate slides prepared and read by two laboratory technicians under microscopes [23].
214 To maintain quality assurance, 10% of the slides were randomly selected and examined by a
215 third technician blinded from the initial results from the other technicians.

# 216 Classification of infection intensity

217 The infection intensity, commonly called worm burden, is typically measured indirectly by
218 counting the number of eggs in faeces, expressed as eggs per gram (epg). This is because
219 indirect methods are more convenient and less invasive [24]. Direct methods of classifying
220 worm burden, which involve counting the number of expelled worms after anti-helminthic
221 treatment, may not be feasible during mass surveys and in areas with low worm burden.
222 WHO classifies infection intensity as light, moderate, or heavy using indirect methods for
223 each type of STH [24]. The epg estimate was applied to categorize the samples based on
224 infection intensity following WHO guidelines. For *A. lumbricoides* infections, light intensity
225 was defined as 1–4,999 epg, moderate intensity as 5,000–49,999 epg, and heavy intensity as
226 ≥50,000 epg. *T. trichiura* infections were classified as light intensity with 1–999 epg,
227 moderate intensity with 1,000–9,999 epg, and heavy intensity with ≥10,000 epg. For
228 hookworm infections, light intensity was defined as 1–1,999 epg, moderate intensity as
229 2,000–3,999 epg, and heavy intensity as ≥4,000 epg.

# 230 Data collection and management

231 Data were collected via mobile phone using Open Data Kit [ODK] and uploaded to a central
232 secure server. Three data sets [individual, household, and laboratory] were downloaded from
233 the server and saved on computers at the Ministry of Health Kenya. Using Microsoft Excel,
234 the data were cleaned, and the household and individual files were merged with unique
235 household identification numbers and later with the laboratory file's unique individual
236 identification numbers.

# 237 Data analysis

238 The cleaned dataset was imported into SAS version 9.04 and R version 4.13 to manage and analyze the
239 data.
240 In line with the ICSPM survey methodology, the point prevalence and intensity of STH
241 infections were estimated separately for each parasite species and stratified by county and risk
242 group. We reported one-sided upper 95% confidence intervals (U95%CI) as the survey was
6. designed to determine the lowest threshold under which true prevalence lies.

# 243 Results

244 **Characteristics of Survey population**
245 The survey teams visited 60 villages/clusters in the two counties, with 30 surveyed in each
246 Bomet and Narok. However, due to an electronic data collection issue, participant
247 information from one village in Narok County was lost. A total of 3,348 (48% male, 51%
248 female and 1% unreported) individuals were enrolled in the surveys, of whom 2,335 (69.7%)
249 provided stool samples (Table 1).
250 Among the 2,096 children providing a stool sample, 1,034 (49.3%) were female, and
251 the median age was 3 years for PSAC (interquartile range [IQR]:2–4), 9 years for SAC
252 (IQR:7–12), and 29 years for WRA (IQR: 20–40).

254

255

256

257

258

259 **Table 1. Number of clusters, households, and individuals surveyed by country and risk**
260 **group, Bomet and Narok Counties, 2021**

| **County** | **Clusters^*^** | | **Households** | | **Individuals** | | | |
| --- | --- | --- | --- | --- | --- | --- | --- | --- |
|  | **Sur- veyed**  **n** | **House- holds per cluster**  **mean (SD)** | **En- rolled**  **n** | **Enrolled households where ≥ 1 member provided a stool sample**  **n (%)** | **Risk group** | **En- rolled**  **n** | **Among those enrolled, number of individuals providing stool sample**  **n (%)** | **Among those providing stool sample, number female**  **n (%)** |
| **Bomet** | 30 | 21.8  (6.0) | 654 | 530 (81.0 | **PSAC** | 508 | 361 (71.1) | 186 (51.5) |
|  |  |  |  |  | **SAC** | 1,014 | 749 (73.9) | 387 (51.7) |
|  |  |  |  |  | **WRA** | 225 | 148 (65.8) | 148 (100.0%) |
| **Narok** | 29^†^ | 18.9  (6.3) | 547 | 429 (78.4) | **PSAC** | 554 | 367 (66.2) | 186 (50.1) |
|  |  |  |  |  | **SAC** | 908 | 619 (68.2) | 275 (44.4) |
|  |  |  |  |  | **WRA** | 139 | 91 (65.5 | 91 (100.0) |

*n–Count; %-prevalence; PSAC–preschool-aged children (2–4 years old); SAC–school-aged children (5–14 years old); SD–Standard Deviation; WRA–women of reproductive age (15–49 years old)*

*^*^Clusters are typically a single village; however, in some cases, villages are combined if the projected village size in households is less than the target segment size.*

*^†^Information from surveys in one cluster was lost due to an electronic data collection issue. Therefore, only information on 29 clusters was included in the analysis.*

261

# 262 Prevalence of infection

263 The prevalence of infections appeared to be slightly higher in Bomet County than in Narok
264 County across all risk groups (Table 2 and Fig 2). Among children (PSAC and SAC), the
265 estimated prevalences ranged from 11% to 13% in Narok County and approximately 16% in
266 Bomet County. Among WRA, in both counties, the prevalence of any STH infections was less
267 than 10%, with Narok at 6.6% and Bomet at 8.1%.
268 In both counties, the prevalence of MHI infection among children was above the 2%
269 threshold for elimination as a public health problem. Children in Bomet County appeared to
270 have higher MHI infection prevalence among both PSAC (6.1%) and SAC (5.5%) than
271 corresponding risk groups in Narok County. WRA in Bomet County recorded the lowest
272 prevalence (0.7%) among all risk groups.

# 273 Parasite type

274 In both counties, *A. lumbricoides* caused the most infections, followed by *T. trichura.*
275 Hookworm infections were not detected in Bomet County and were uncommon in Narok
276 County. In Bomet County, the prevalence of *A. lumbricoides* infections was slightly higher
277 among SAC, followed by PSAC, then WRA. In Narok County, *T. trichura* infections
278 followed this pattern; however, unlike Bomet County, the prevalence of *A. lumbridcoides*
279 was shown to be higher among PSAC than SAC.
280 **Table 2. Estimated prevalence of soil-transmitted helminth infection by county, species,**
281 **intensity, and risk group, Bomet and Narok Counties, 2021**

| **County** | **Risk group** | **Providing stool samples**  **n** | **Hookworm^*^**  **% (95% UCL)** | ***A. lumbricoides***  **% (95% UCL)** | ***T. trichiura***  **% (95% UCL)** | **Any STH**  **% (95% UCL)** |
| --- | --- | --- | --- | --- | --- | --- |
| **Any intensity infections** | | | | | | |
| **Bomet** | **PSAC** | 361 | 0.0 | 10.8 (16.1) | 6.4 (10.3) | 15.8 (21.7) |
|  | **SAC** | 749 | 0.0 | 11.3 (15.9) | 8.4 (15.2) | 16.2 (23.1) |
|  | **WRA** | 148 | 0.0 | 6.1 (11.9) | 4.1 (9.4) | 8.1 (14.5) |
| **Narok** | **PSAC** | 367 | 0.3 (1.2) | 9.5 (14.5) | 4.6 (7.2) | 12.8 (18.2) |
|  | **SAC** | 619 | 0.3 (1.5) | 6.3 (10.0) | 6.8 (10.7) | 11.3 (16.2) |
|  | **WRA** | 91 | 1.1 (5.1) | 4.4 (10.8) | 2.2% (6.7%) | 6.6 (13.3) |
| **Moderate-to-heavy intensity infections** | | | | | | |
| **Bomet** | **PSAC** | 361 | 0.0 | 5.8 (9.3) | 0.8 (2.1) | 6.1 (9.5) |
|  | **SAC** | 749 | 0.0 | 5.5 (8.7) | 0.1 (0.6) | 5.5 (8.7) |
|  | **WRA** | 148 | 0.0 | 0.7 (3.2) | 0.0 | 0.7 (3.2) |
| **Narok** | **PSAC** | 367 | 0.0 | 2.7 (5.7) | 1.1 (2.9) | 3.3 (6.3) |
|  | **SAC** | 619 | 0.0 | 2.4 (4.3) | 0.5 (1.6) | 2.7 (4.7) |
|  | **WRA** | 91 | 0.0 | 3.3 (8.0) | 0.0 | 3.3 (8.0) |

*n–Count;%–Prevalence; UCL–Upper 95% one-sided confidence limit; PSAC–preschool-aged children (2–4 years old); SAC–school-aged children (5–14 years old); STH–Soil-transmitted helminth; WRA–women of reproductive age (15–49 years old)*

*^*^N. americanus and A. duodenale*

282

283 **<<<Figure 2>>>>**
284 **Fig 2. Estimated prevalence of soil-transmitted helminth infection by county, species,**
285 **intensity, and risk group, Bomet and Narok Counties, 2021**
286 *^*^N. americanus and A. duodenale*

287

# 288 Co-infections

289 Co-infections were uncommon (Table 3). When identified, they were most frequently
290 *A.lumbricoides* and *T.trichura,* with all risk groups in both counties recording at least one co-
291 infection. No triple infections were detected.
292 **Table 3. Estimated soil-transmitted helminth coinfection prevalence by county and risk**
282 **group, Bomet and Narok Counties, 2021**

| **Coun ty** | **Risk grou p** | **provi ng stool samp les**  **n** | **Uninfec ted**  **n (%)** | **Mono-**  **infecti on** | | | **Co-**  **infectio n** | | | **Triple**  **infectio n** |
| --- | --- | --- | --- | --- | --- | --- | --- | --- | --- | --- |
|  |  |  |  | **Hookw orm^*^**  **n (%)** | ***A.***  ***lumbri***  ***-coides***  **n (%)** | ***T.***  ***trichiu ra***  **n (%)** | **Hookw orm and *A.***  ***lumbri- coides***  **n (%)** | **Hookw orm and**  ***T.***  ***trichiur a***  **n (%)** | ***A.***  ***lumbri***  ***-coides* and *T.***  ***trichiu ra***  **n (%)** | **Hookwo rm, *A. lumbric oides*, and *T. trichiura***  **n (%)** |
|  | **PSA** | 361 | 304 | 0 | 34 | 18 | 0 (0.0) | 0 | 5 | 0 (0.0) |
|  | **C** |  | (84.2) | (0.0) | (9.4) | (5) |  | (0.0) | (1.4) |  |
| **Bom** | **SAC** | 749 | 628 | 0 | 58 | 36 | 0 (0.0) | 0 | 27 | 0 (0.0) |
| **et** |  |  | (83.8) | (0.0) | (7.7) | (4.8) |  | (0.0) | (3.6) |  |
|  | **WR** | 148 | 136 | 0 | 6 | 3 | 0 (0.0) | 0 | 3 | 0 (0.0) |
|  | **A** |  | (91.9) | (0.0) | (4.1) | (2.0) |  | (0.0) | (2.0) |  |
|  | **PSA** | 367 | 320 | 0 | 29 | 12 | 1 (0.3) | 0 | 5 | 0 (0.0) |
|  | **C** |  | (87.2) | (0.0) | (7.9) | (3.3) |  | (0.0) | (1.4) |  |
| **Naro** | **SAC** | 619 | 549 | 1 | 27 | 29 | 0 (0.0) | 1 | 12 | 0 (0.0) |
| **k** |  |  | (88.7) | (0.2) | (4.4) | (4.7) |  | (0.2) | (1.9) |  |
|  | **WR** | 91 | 85 | 1 | 3 | 1 | 0 (0.0) | 0 | 1 | 0 (0.0) |
|  | **A** |  | (93.4) | (1.1) | (3.3) | (1.1) |  | (0.0) | (1.1) |  |

*n–Count; %–Prevalence; PSAC–preschool-aged children (1–4 years old); SAC–school-aged children (5–14 years old); WRA–women of reproductive age (15–49 years old)*

*^*^N. americanus and A. duodenale*

294

# 295 Discussion

296 Our findings underscore persistent transmission of STH, evidenced by moderate (10–<20%)
297 STH infection prevalence among children in Bomet and Narok Counties. Prevalence
298 estimates of any STH infection were above the 2% treatment threshold target according to the
299 breaking transmission strategy [12], and the prevalence of MHI infections also exceeded the
300 WHO 2030 target for elimination as a public health problem (<2%) among all surveyed
301 children [7]. These findings are consistent with recent findings from geostatistical modelling
302 of STH using a nationwide survey dataset that showed a high probability of sub counties in
303 the southwestern part of Kenya where Narok and Bomet are located exceeding the 2% and
304 10% prevalence threshold for *A.lumbricoides* and *T.trichura*[35].
305 These results are critical for informing programmatic decisions, particularly on the
306 continuation of MDAs. While the 2% threshold is an established benchmark for guiding such
307 interventions, its interpretation must consider local transmission dynamics[33,34]. Similar
308 patterns of persistent infection despite continued PC among SAC have been highlighted in
309 other studies, reflecting the relative slow decline of infections despite continued school based
310 deworming[37,38]. These findings suggest that tailoring of continued annual PC in these
311 counties is required to make progress toward the elimination of STH morbidity as a public
312 health problem in the country.
313 Compared to prior surveys, the estimated prevalences among SAC from our
314 community-based surveys (Bomet, 16.2%, and Narok, 11.3%) were lower than those
315 demonstrated by school-based impact surveys conducted in 2017, five years after the
316 commencement of the National School-Based Deworming Programme. These 2017 surveys
317 showed a reduction of STH among SAC in Bomet from 29.7% in 2012 to 18.1% in 2017 and,
318 in Narok, from 53% in 2012 to 41% in 2017[15]. However, the prevalences are higher than
319 those derived from the ten-year model based geostatistical impact surveys (Bomet 8.3%, and
320 Narok 10.8%) conducted between 2021 and 2022 [25]. Despite there being an overall decline
321 in the prevalence of STH from baseline in the country, the rate of decline in these two
322 counties has been relatively lower, which could be explained by poor sanitation facilities,
323 resulting in possible re-infection [26].

324

# 325 Parasite species

326 *A. lumbricoides* was the most common STH infection observed in the two counties,
327 followed by *T. trichura,* with very few hookworm infections detected. This finding
328 corresponds with school-based surveys conducted by the National School-based Deworming
329 Programme, which found *A. lumbricoides* to be the most common STH during baseline,
330 midline, and end-line surveys in the country as well as geostatistical modelling that showed
331 *A.lumbricoides* and *T.trichura* to be prevalent in these areas [17,35]. This may be due to
332 helminths' long lifespan in human hosts and their production of many environmentally
333 resistant eggs, which are expelled in faeces to enhance their survival and lifecycle
334 continuation, leading to reinfection [16,27].
335 The survey also concurs with findings from the National School-based Deworming
336 Programme that did not show significant reductions in the prevalence of MHI infection with
337 *T. trichura,* where albendazole is the drug distributed. In contrast, it has been shown that a
338 combination of ivermectin and albendazole would be the drug of choice in areas with high *T.*
339 *trichura* infections[26]*,* and WHO now suggests including ivermectin in addition to
340 albendazole in such settings[44]. Drugs such as emodepside are also currently being tested in
341 humans and show great promise as alternative treatments to address the limited effectiveness
342 of benzimidazoles against *T. trichiura*[45] *.*
343 It is also possible that reinfection occurs among the at-risk age groups, resulting in a
344 resurgence of prevalence, as stated in a meta-analysis that showed infection after treatment
345 occurs rapidly, particularly for *A. lumbricoides* and *T.trichura* [16].

# 346 Risk groups targeted by PC

347 Both infection prevalence and MHI infection prevalence were similar among PSAC and SAC
348 within both counties. The consistency in high prevalence among PSACs who are routinely
349 excluded from PC distributions highlights the fact that they should be considered for
350 targeting during deworming exercises, as infections among this age group may lead to
351 morbidity and cognitive impairment and may compromise the performance of PSAC on
352 expressive language [27].
353 In addition, given the prevalence of similar magnitude among PSAC and SAC, the
354 Ministry may consider expanding beyond school-based PC to include other at-risk groups,
355 such as PSAC and WRA, to achieve broader community impact, as shown in studies from
356 western and coastal Kenya and Malawi[35,43,44]. This expansion beyond SAC may enable
357 the country to accelerate progress toward eliminating STH as a public health problem in line
358 with the Breaking Transmission Strategy and the WHO 2030 NTD Road Map, similar to the
359 approach taken by Kenya to conduct community wide mapping in the coastal part of Kenya,
360 coupled with higher coverage rates and the ability of reaching non-enrolled SAC through
361 community wide MDAs[39,50].

# 362 Limitations

363 The survey was designed to estimate prevalence based on county-level administrative
364 boundaries. However, intra-county prevalence heterogeneity is likely. Hence, the program
365 would not be able to consider the diversity of the ecological conditions that have implications
366 on the drivers of STH transmission within the counties, contributing to the heterogeneous
367 classification of infection. This, in turn, would affect decision-making as to where PC
368 distribution would be most efficient and cost-effective for the program. Subsequently, with
369 these results, PC administration would be conducted in the entire county and not in specific
370 focal areas with a prevalence above 2%, contrary to the BTS, which stipulates that the
371 implementation unit for treatment is the ward.
372 The sensitivity of the Kato Katz technique in detecting hookworm eggs is reduced in
373 low-prevalence settings and with sample transportation delays between the time of collection
374 in the field and analysis in the laboratory. Even under optimal conditions, its diagnostic
375 sensitivity remains low for hookworm, with studies showing that this method may
376 substantially underestimate true infection prevalence[32]. To mitigate this risk, laboratories
377 were established at the nearest health centres where teams were conducting the survey. This
378 significantly reduced the time between sample collection and laboratory analysis. Delays in
379 sample shipment would lead to an underestimation of the true prevalence of hookworm
380 infections in the sampled population[30,31]. In addition, some participants who were
381 provided with stool pots didn’t provide samples. However, the number was not significant as
382 the sample size for the study was still achieved.
383 During the survey conducted in Narok County, data from one village was lost,
384 reducing the sample size. This loss may have influenced the STH prevalence estimate in the
385 county. However, it's anticipated that any shifts in the county-level prevalence would likely
386 be minimal given that data from the missing village represent approximately 1/30th of the
387 total sample size.

# 388 Conclusion

389 Our survey showed that STH infections remain a public health problem in Narok and
390 Bomet counties. The elevated infection prevalence and MHI infection prevalence among all
391 surveyed groups, including PSAC and WRA, suggest that expansion to risk groups beyond
392 SAC during deworming exercises, as envisioned in the Breaking Transmission Strategy, may
393 accelerate progress to controlling STH infections.

# 394 Acknowledgments

395 We thank the children and parents who participated in this survey, and the supervision
396 and technical support from the Division of Vector-Borne and Neglected Tropical Diseases,
397 Children Without Worms, and the Africa Field Epidemiology Network [AFENET]. We are
398 grateful to the field officers who collected the data and the data team who worked tirelessly to
399 ensure the survey was completed. We acknowledge the support from the national and county
400 management in Bomet and Narok counties.

401

402 **Disclosure:** This research was funded by a grant provided to Children Without Worms (Georgia, USA) by GSK
403 (United Kingdom) to support surveys conducted by the Division of Vector-Borne and Neglected Tropical
404 Diseases, Ministry of Health, Kenya. The funders had no role in the survey design, data collection, data analysis,
405 or interpretation. The findings and conclusions of the survey reflect the view of the authors only.
406 **Data Availability Statement:** The data used for the analyses in this paper are owned by the government of
407 Kenya, and are subject to provisions of the Data Protection Act
408 <https://kenyalaw.org/kl/fileadmin/pdfdownloads/LegalNotices/2021/LN263_2021.pdf> . Researchers are
409 welcome to request access to de-identified datasets from the Division of Vector-Borne and Neglected Tropical
410 Diseases at the Ministry of Health [(headdvbntd@gmail.com).](mailto:(headdvbntd@gmail.com)
411 **Competing Interests**: I have read the journal’s policy and the authors of the manuscript have the following
412 competing interests: KMS receives a salary from The Task Force for Global Health, an organization that
413 receives funding from GSK and Johnson & Johnson, the manufacturers of albendazole and mebendazole,
414 respectively.
415 **Basemap Information:**
416 •	Kenya - Subnational Administrative Boundaries (<https://data.humdata.org/dataset/cod-ab-ken>)
417 •	License: [Creative Commons Attribution for Intergovernmental Organisations](https://data.humdata.org/faqs/licenses)

418

419

420

421

422

423

# 424 References

425 1.	WHO. Ending the neglect to attain the Sustainable Development Goals: a road map for
426 neglected tropical diseases 2021–2030. Geneva: World Health Organization
427 (https://[www.who.int/neglected_diseases/Revised-DraftNTD-Roadmap-](http://www.who.int/neglected_diseases/Revised-DraftNTD-Roadmap-)
428 23Apr2020.pdf). Word Health Organization. 2020. 196 p.
429 2.	Id R, Mpanya A, Inocencio R, Id SL, Id EH, Boelaert M. Community-based survey on
430 helminth infections in Kwilu province , the Democratic Republic of the Congo , and
431 implications for local control strategies. 2020;1–14.
432 3.	Brooker S. Estimating the global distribution and disease burden of intestinal
433 nematode infections : Adding up the numbers – A review. Int J Parasitol [Internet].
434 2010;40(10):1137–44. Available from: <http://dx.doi.org/10.1016/j.ijpara.2010.04.004>
435 4.	Chen J, Gong Y, Chen Q, Li S, Zhou Y. Global burden of soil-transmitted helminth
436 infections, 1990–2021. Infect Dis Poverty. 2024 Dec 1;13(1).
437 5.	Jourdan PM, Lamberton PHL, Fenwick A, Addiss DG. Seminar Soil-transmitted
438 helminth infections. The Lancet. 2017;6736(17):1–14.
439 6.	Bethony J, Brooker S, Albonico M, Geiger SM, Loukas A, Diemert D, et al. Soil-
440 transmitted helminth infections : ascariasis , trichuriasis , and hookworm. 2006;367.
441 7.	World Health Organization (WHO). 2030 targets for soil-transmitted helminthiases
442 control programmes. 2012;
443 8.	Pullan RL, Gething PW, Smith JL, Mwandawiro CS, Sturrock HJW, Gitonga CW, et
444 al. Spatial Modelling of Soil-Transmitted Helminth Infections in Kenya : A Disease
445 Control Planning Tool. 2011;5(2).
446 9.	Ministry of Health Kenya. The 2nd Kenya National Strategic Plan For control of
447 NEGLECTED TROPICAL DISEASES 2016-2020. 2020;
448 10.	Ng’ang’a M, Matendechero S, Kariuki L, Omondi W, Makworo N, Owiti PO, et al.
449 Burden of Soil Transmitted Helminthiasis in Primary school children in Migori
450 County, Kenya. Supplement) East African Medical Journal. 2016;93(10):32–9.
451 11.	Ministry of Health Kenya. The 2nd Kenya National Strategic Plan For control of
452 Neglected Tropical Diseases. 2015.
453 12.	Ministry of Health Kenya. THE KENYA NATIONAL BREAKING
454 TRANSMISSION STRATEGY FOR SOIL-TRANSMITTED HELMINTHIASIS,
455 SCHISTOSOMIASIS, LYMPHATIC FILARIASIS AND TRACHOMA. 2019;
456 13.	Anderson RM, Turner HC, Truscott JE, Hollingsworth TD. Should the Goal for the
457 Treatment of Soil Transmitted Helminth ( STH ) Infections Be Changed from
458 Morbidity Control in Children to Community-Wide Transmission Elimination ?
459 2020;1–8.
460 14.	Okoyo C, Nikolay B, Kihara J, Simiyu E, Garn J V., Freeman MC, et al. Monitoring
461 the impact of a national school based deworming programme on soil-transmitted
462 helminths in Kenya: The first three years, 2012 - 2014. Parasit Vectors. 2016;9(1):1–
463 13.
464 15.	Mwandawiro C, Okoyo C, Kihara J, Simiyu E, Kepha S, Campbell SJ, et al. Results of
465 a national school-based deworming programme on soil-transmitted helminths
466 infections and schistosomiasis in Kenya : 2012 – 2017. 2019;1–18.
467 16.	King CH, Zhou X nong, Jia T wu, Melville S. Soil-Transmitted Helminth Reinfection
468 after Drug Treatment : A Systematic Review and Meta-Analysis. 2012;6(5).
469 17.	Njenga SM, Mwandawiro CS, Muniu E, Mwanje MT, Haji FM, Bockarie MJ. Adult
470 population as potential reservoir of NTD infections in rural villages of Kwale district ,
471 Coastal Kenya : implications for preventive chemotherapy interventions policy.
472 2011;2–7.
473 18.	Njenga SM, Mutungi FM, Wamae CN, Mwanje MT, Njiru KK, Bockarie MJ. Once a
474 year school-based deworming with praziquantel and albendazole combination may not
475 be adequate for control of urogenital schistosomiasis and hookworm infection in
476 Matuga District , Kwale County , Kenya. 2014;1–9.
477 19.	Pullan RL, Halliday KE, Oswald WE, Mcharo C, Beaumont E, Kepha S, et al. Effects,
478 equity, and cost of school-based and community-wide treatment strategies for soil-
479 transmitted helminths in Kenya: a cluster-randomised controlled trial. The Lancet.
480 2019 May 18;393(10185):2039–50.
481 20.	Anderson RM, Turner HC, Truscott JE, Hollingsworth TD. Should the Goal for the
482 Treatment of Soil Transmitted Helminth ( STH ) Infections Be Changed from
483 Morbidity Control in Children to Community-Wide Transmission Elimination ?
484 2020;1–8.
485 21.	Halliday KE, Oswald WE, McHaro C, Beaumont E, Gichuki PM, Kepha S, et al.
486 Community-level epidemiology of soiltransmitted helminths in the context of school-
487 based deworming: Baseline results of a cluster randomised trial on the coast of Kenya.
488 PLoS Negl Trop Dis [Internet]. 2019;13(8):1–22. Available from:
489 <http://dx.doi.org/10.1371/journal.pntd.0007427>
490 22.	Narok County Government. Narok County Integrated Development Plan 2018-2022.

491	2022;1–52.

492	23.	County Government of Bomet. County Government of Bomet County Integrated

493	Development Plan 2018-2022. 2018;(February 2018):518.

494 24.	Tupps C, Kargbo-Labour I, Paye J, Dhakal S, Hodges MH, Jones AH, et al.
495 Community-wide prevalence and intensity of soil-transmitted helminthiasis and
496 Schistosoma mansoni in two districts of Sierra Leone. PLoS Negl Trop Dis. 2022 May
497 1;16(5).
498 25.	Davlin SL, Jones AH, Tahmina S, Kawsar A Al, Joshi A, Zaman SI, et al. Soil-
499 transmitted helminthiasis in four districts in Bangladesh: Household cluster surveys of
500 prevalence and intervention status. BMC Public Health. 2020 May 12;20(1).
501 26.	Tinkitina B, Beinamaryo P, Kyarisiima H, Nabatte B, Arinaitwe M, Mubangizi A, et
502 al. Prevalence and intensity of soil-transmitted helminth infections among school-aged
503 children in five districts in Uganda. PLoS Negl Trop Dis. 2024 Aug 1;18(8).
504 27.	Kenya National Bureau of Statistics. Kenya Population and Housing Census 2019

505	Volume III. Vol. 21. 2020. 1–9 p.

506 28.	Jones A. Integrated Community-Based Survey for Program Monitoring (ICSPM)
507 Reference Manual, version 2.2 Integrated Community-Based Survey for Program
508 Monitoring, version 2.2 ii incorporates input from CWW staff on lessons learned in the
509 first ICSPMs in Bangladesh. adds Annexes 12 and 13 and incorporates comments adds
510 selection probabilities to Annex 4 for SAC and WRA. This manual and other program
511 tools prepared by CWW can be downloaded at childrenwithoutworms.org/icspm-
512 manual-resources. 2017.
513 29.	World Health Organization. Assessing the epidemiology of soil-transmitted helminths
514 during a transmission assessment survey (TAS) [Internet]. 2015 [cited 2025 Apr 26].
515 Available from: https://[www.who.int/publications/i/item/9789241508384](http://www.who.int/publications/i/item/9789241508384)
516 30.	Nikolay B, Brooker SJ, Pullan RL. Sensitivity of diagnostic tests for human soil-
517 transmitted helminth infections: A meta-analysis in the absence of a true gold standard.
518 Int J Parasitol. 2014 Oct 1;44(11):765–74.
519 31.	Katz N, Chaves A, Pellegrino J. A simple device for quantitative stool thick-smear
520 technique in Schistosomiasis mansoni. Rev Inst Med Trop Sao Paulo. 1972;14(6):397–
521 400.
522 32.	World Health Organization (WHO). Preventive chemotherapy guideline to control
523 soil-transmitted helminth infections in at-risk population groups. 2017.
524 33.	Mutono N, Njuguna B, Kepha S, Wakesho F, Omondi W, Kibati P, et al. Geostatistical
525 modelling of soil-transmitted helminth prevalence in Kenya: Informing targeted
526 interventions to accelerate elimination efforts. International Journal of Infectious
527 Diseases. 2025 Jul 1;156.
528 34.	Werkman M, Wright JE, Truscott JE, Easton A V., Oliveira RG, Toor J, et al. Testing
529 for soil-transmitted helminth transmission elimination: Analysing the impact of the
530 sensitivity of different diagnostic tools. PLoS Negl Trop Dis. 2018 Jan 1;12(1).
531 35.	Truscott JE, Hardwick RJ, Werkman M, Saravanakumar PK, Manuel M, Ajjampur
532 SSR, et al. Forecasting the effectiveness of the DeWorm3 trial in interrupting the
533 transmission of soil-transmitted helminths in three study sites in Benin, India and
534 Malawi. Parasit Vectors. 2021 Dec 1;14(1).
535 36.	Masaku J, Okoyo C, Araka S, Musuva R, Njambi E, Njomo DW, et al. Understanding
536 factors responsible for the slow decline of soil-transmitted helminthiasis following
537 seven rounds of annual mass drug administration (2012–2018) among school children
538 in endemic counties of Kenya: A mixed method study. PLoS Negl Trop Dis. 2023 May
539 1;17(5).
540 37.	Burnim M, Ivy JA, King CH. Systematic review of community-based, school-based,
541 and combined delivery modes for reaching school-aged children in mass drug
542 administration programs for schistosomiasis. PLoS Negl Trop Dis. 2017 Oct
543 27;11(10).
544 38.	Okoyo C, Minnery M, Orowe I, Owaga C, Wambugu C, Olick N, et al. Using a model-
545 based geostatistical approach to design and analyse the prevalence of schistosomiasis
546 in Kenya. 2023;(November):1–10.
547 39.	Masaku J, Okoyo C, Araka S, Musuva R, Njambi E, Njomo DW, et al. PLOS
548 NEGLECTED TROPICAL DISEASES Understanding factors responsible for the slow
549 decline of soil-transmitted helminthiasis following seven rounds of annual mass drug
550 administration ( 2012 – 2018 ) among school children in endemic counties of Kenya :
551 A mixe. 2023;1–29.
552 40.	Flammer PG, Smith AL, Smith AL. Intestinal helminths as a biomolecular complex in
553 archaeological research. 2020;
554 41.	Hürlimann E, Keller L, Patel C, Welsche S, Hattendorf J, Ali SM, et al. Efficacy and
555 safety of co-administered ivermectin and albendazole in school-aged children and
556 adults infected with Trichuris trichiura in Côte d’Ivoire, Laos, and Pemba Island,
557 Tanzania: a double-blind, parallel-group, phase 3, randomised controlled trial. Lancet
558 Infect Dis. 2022;22(1):123–35.
559 42.	World Health Organization. Assessing schistosomiasis and soil-transmitted
560 helminthiases control programmes Monitoring and evaluation framework. 2024.
561 43.	Krücken J, Holden-Dye L, Keiser J, Prichard RK, Townson S, Makepeace BL, et al.
562 Development of emodepside as a possible adulticidal treatment for human
563 onchocerciasis-The fruit of a successful industrial-academic collaboration. Vol. 17,
564 PLoS Pathogens. Public Library of Science; 2021.
565 44.	Mazibuko XI, Chimbari M. The effect of schistosomiasis and soil-transmitted
566 helminths on expressive language skills among African preschool children. BMC
567 Infect Dis. 2022;22(1):1–9.
568 45.	Makaula P, Kayuni SA, Mamba KC, Bongololo G, Funsanani M, Juziwelo LT, et al.
569 Mass drug administration campaigns: comparing two approaches for schistosomiasis
570 and soil-transmitted helminths prevention and control in selected Southern Malawi
571 districts. BMC Health Serv Res. 2024 Dec 1;24(1).
572 46.	Mwinzi PNM, Montgomery SP, Owaga CO, Mwanje M, Muok EM, Ayisi JG, et al.
573 Integrated community-directed intervention for schistosomiasis and soil transmitted
574 helminths in western Kenya - A pilot study. Parasit Vectors. 2012;5(1).
575 47.	Kepha S, Ochol D, Wakesho F, Omondi W, Njenga SM, Njaanake K, et al. Precision
576 mapping of schistosomiasis and soil-transmitted helminthiasis among school age
577 children at the coastal region, Kenya. PLoS Negl Trop Dis [Internet].
578 2023;17(1):e0011043. Available from: <http://dx.doi.org/10.1371/journal.pntd.0011043>
579 48.	Loukas A, Hotez PJ, Diemert D, Yazdanbakhsh M, Correa-oliveira R, Croese J.
580 Hookworm infection. 2016;
581 49.	Bosch F, Palmeirim MS, Ali SM, Ame SM, Hattendorf J, Keiser J, et al. Diagnosis of
582 soil-transmitted helminths using the kato-katz technique: What is the influence of
583 stirring, storage time and storage temperature on stool sample egg counts? PLoS Negl
584 Trop Dis. 2021;15(1):1–17.

585

586

Figure	[Click here to access/download;Figure;Fig 1.tif](https://www2.cloud.editorialmanager.com/pntd/download.aspx?id=1474873&guid=636a3417-d506-49bf-be29-b4847adf8082&scheme=1)[<https://www2.cloud.editorialmanager.com/pntd/download.aspx?id=1474873&guid=636a3417-d506-49bf-be29-b4847adf8082&scheme=1>](https://www2.cloud.editorialmanager.com/pntd/download.aspx?id=1474873&guid=636a3417-d506-49bf-be29-b4847adf8082&scheme=1)

Figure	[Click here to access/download;Figure;Fig2.tif](https://www2.cloud.editorialmanager.com/pntd/download.aspx?id=1474874&guid=3c304a07-f82c-4225-8501-fdd2144aa205&scheme=1)[<https://www2.cloud.editorialmanager.com/pntd/download.aspx?id=1474874&guid=3c304a07-f82c-4225-8501-fdd2144aa205&scheme=1>](https://www2.cloud.editorialmanager.com/pntd/download.aspx?id=1474874&guid=3c304a07-f82c-4225-8501-fdd2144aa205&scheme=1)

Response to Reviewers

**Date: 12th August 2025**

**Dear Reviewers**

We thank you and the reviewers for the thoughtful and constructive feedback provided. Below, we address each of the reviewers' comments, providing clarifications, justifications, and detailing revisions made to the manuscript.

**Reviewers' comments:**

**Reviewer's Responses to Questions**

**Key Review Criteria Required for Acceptance?**

**As you describe the new analyses required for acceptance, please consider the following:**

**Methods**

**-Are the objectives of the study clearly articulated with a clear testable hypothesis stated?**

**-Is the study design appropriate to address the stated objectives?**

**-Is the population clearly described and appropriate for the hypothesis being tested?**

**-Is the sample size sufficient to ensure adequate power to address the hypothesis being tested?**

**-Were correct statistical analysis used to support conclusions?**

**-Are there concerns about ethical or regulatory requirements being met?**

**Reviewer #1: i) Are the objectives of the study clearly articulated with a clear testable hypothesis stated?**

**Acceptable, Even if the link with the hypothesis is not very clear.**

ii) **Is the study design appropriate to address the stated objectives? Yes, But some shortcomings in applicability.**
iii) **Is the population clearly described and appropriate for the hypothesis being tested?**

**Yes**

iv) **Is the sample size sufficient to ensure adequate power to address the hypothesis being tested?**

**approximately**

We thank the reviewers for their insights into sample size calculations. As per the ICSPM manual, the sample size of 332 per risk group was calculated to ensure that, even with this design effect, we could classify prevalence thresholds with adequate statistical power, particularly for determining whether prevalence was below key programmatic cutoffs (e.g., <2%, <10%, <20%). This methodology has been highlighted in the ICSPM manual, the WHO manual <https://www.who.int/publications/i/item/9789241508384> and several other publications including <https://journals.plos.org/plosntds/article?id=10.1371/journal.pntd.0011605> .

v) **Were correct statistical analysis used to support conclusions?**

**No statistical tests were actually carried out, just descriptive data. It would be necessary to compare the groups (PSAC, SAC, WRA) and according to demographic parameters (sex, age, etc.).**

We thank the reviewer for this comment. However as per ICSPM methodology and our data analysis plan, we did not assess risk factors in this manuscript. This is because the study was powered to classify prevalence thresholds per risk group, not to detect differences between subgroups

vi) **Are there concerns about ethical or regulatory requirements being met? I would have preferred ethical approval by the National Ethics Committee. Given that this is a national issue.**

We take note of the reviewers’ comments and would like to state that ethical approval was granted by the Amref Health Africa Ethics and Scientific Review Committee, which is accredited by the National Commission for Science, Technology and Innovation (NACOSTI), the national body. At the time of the study, this was deemed sufficient to conduct the study. Future surveys will seek approval from the National Ethics Committee as per updated national requirements.

**Reviewer #2: The methods read more detailed now than last time. I am a bit confused that they are placed at the end of the manuscript, squeezed in between required information for the journal. I think this should be solved and the methods should be placed before the results. Made it a bit hard to jump back and forth between methods and results, too.**

We take note of the comments and have moved the methods section before the results The initial change was due to an editorial comment.

**Reviewer #3: The revised manuscript addresses the main concerns raised in the initial review. The study objectives are clearly stated and appropriate for the public health surveillance context. The cross-sectional design is suitable, and the target population is well defined.**

**Sample size justification is now aligned with ICSPM and WHO guidelines, and the statistical methods used (one-sided U95% CIs, DEFF = 2.0) are appropriate for threshold-based programmatic decisions. Ethical procedures are adequately described, including consent, health education, and referrals for treatment.**

**Overall, the methodology is sound, the data are well analyzed, and the revisions improve the manuscript’s clarity and relevance.**

We thank the reviewer for their comments and appreciate thei insight into improving the clarity of the manuscript.

**Results**

**-Does the analysis presented match the analysis plan?**

**-Are the results clearly and completely presented?**

**-Are the figures (Tables, Images) of sufficient quality for clarity?**

**Reviewer #1: i) Does the analysis presented match the analysis plan? Acceptable with some shortcomings.**

ii) **Are the results clearly and completely presented?**

**Some aspects are missing...see section v of methods. Intra- and extra-group stratification analyses would be necessary. These results could be presented in an appendix, for example!**

We appreciate the comments made by the reviewer. However, the results presented in this paper adhere to the previously established analysis plan as outlined in the ICSPM protocol.

iii) **Are the figures (Tables, Images) of sufficient quality for clarity?**

**Please match the places and dates in the titles of the figures and tables. Figure legends are not marked. Figure numbers do not appear! North is not shown on the map.**

Figure and table numbers have been updated.

**Reviewer #2: The results read fine and are presented in tables and figures. Here some comments on the results:**

- **I think it would be nice to include a flowchart to show the recruitment process in more detail and include i) in how many households was nobody home or no adult/head of household, ii) in how many households, individuals did not want to consent, iii) how many individuals consented but did not provide a stool sample, iv) how many individuals were excluded because they did not fulfil the eligibility criteria such as staying in the household the night before and after.**
- **I think a logistic regression would have been a nice add-on to this publication. I think it is not a must but would give the presentation more substance.**
- **The caption of Figure 2 should include the abbreviation used for the legend of the Figure. I am aware that the heading in the figure itself includes it but for completeness I would include it in the caption or even consider to exchange the HMI abbreviation for the full wording in the figure.**

We take note of the comment from the reviewer and have updated the caption of Figure 2 to match that used in the legend.

**Reviewer #3: The analysis presented is consistent with the ICSPM methodology and aligns with the stated objectives and analysis plan. The results are clearly and comprehensively presented, including stratification by species, risk group, and infection intensity. Figures and tables are of good quality and effectively support the findings. The use of Table 3 to present co- infection patterns is appropriate and sufficiently detailed.**

We appreciate the comments made by the reviwer to improve the readability of the manuscript.

**Conclusions**

**-Are the conclusions supported by the data presented?**

**-Are the limitations of analysis clearly described?**

**-Do the authors discuss how these data can be helpful to advance our understanding of the topic under study?**

**-Is public health relevance addressed?**

**Reviewer #1: i) Are the conclusions supported by the data presented?**

**Yes**

ii) **Are the limitations of analysis clearly described? Not really!**
iii) **Do the authors discuss how these data can be helpful to advance our**

**understanding of the topic under study?**

**Yes, but a few more sentences would be necessary!**

v) **Is public health relevance addressed?**

**You could put it this way. Authors should highlight the importance of the study to the public and the scientific community.**

**Reviewer #2: The discussion needs a bit more discussion. Two examples:**

1. **The authors write: "Despite there being an overall decline in the prevalence of STH from baseline in the country, the rate of decline in these two counties has been relatively lower, which could be explained by poor sanitation facilities, resulting in possible re-infection[26]." -- So the prevalence declined but not as much and you give poor sanitation facilities as the reason. I would suggest to discuss how these could be improved. Were they already improved a little bit, therefore the decline? Where does the decline come from if its not the sanitation facilities? What would the counties need to improve them, of who is this the responsibility? Also, how did other countries manage to bring the prevalence down? Was it also because of WASH facilities or are there any other ways to reduce prevalence?**

We acknowledge the reviewers request to flesh out the discussion regarding the overall decline in prevalence. We have included additional information from geostatistical modelling that highlights some of the reasons that concur with this finding.

2. **The authors write: "In contrast, it has been shown that a combination of ivermectin and albendazole would be the drug of choice in areas with high T. trichura infections[26]. Additionally, it has been shown that there is heterogeneity of STH transmission, which would necessitate a different approach in areas found to have high T. trichuris infections.(41). It is also possible that reinfection occurs among the at-risk age groups, resulting in a resurgence of prevalence, as stated in a meta-analysis that showed infection after treatment occurs rapidly, particularly for A. lumbricoides and T.trichura [16]." -- This paragraph reads incomplete to me. You write about a drug combination but you do not discuss how this might impact the prevalence in your setting. You write about heterogeneity and a different approach but you do not mention the approach or what this would mean for your counties or elsewhere. And you write about reinfections but you do not discuss how they could be prevented.**

We thank the reviewer for this comment. We have added information on the impact of deworming programs on the prevalence of infection as well as mitigation measures on how to prevent reinfection using WASH and behaviour change strategies.

**Reviewer #3: The conclusions are well supported by the data presented. The manuscript clearly describes limitations, including diagnostic sensitivity and geographic heterogeneity. The discussion effectively contextualizes findings within broader regional experiences and outlines implications for expanding**

**preventive chemotherapy. The public health relevance is well articulated, particularly regarding policy decisions to reach underserved risk groups and sustain progress toward STH elimination targets.**

**Editorial and Data Presentation Modifications?**

**Use this section for editorial suggestions as well as relatively minor modifications of existing data that would enhance clarity. If the only modifications needed are minor and/or editorial, you may wish to recommend “Minor Revision” or “Accept”.**

**Reviewer #1: Authors should review the order of the writing plan! The "Methods" section appears after the acknowledgments... I don't know if this is a requirement of the editorial line!**

We thank the reviewer for this observation. The editorial team had suggested the movement of the methods section to the end which has now been reversed for easier reading.

**Reviewer #2: lines 76-79: Reference number 3 is from 2010 and therefore rather old. And reference number 4 is referring to a study solely conducted in Ethiopia. It does not seem to be a good fit for the sentence here. What about the WHO weekly epidemiological report from 2024?**

We thank the reviewer for this important information. We have included the population requiring PC and those treated within the WHO African region from the WHO weekly epidemiological record in the manuscript in line 90-92.

**line 88 and many others: There is no consistency in presenting the references. Sometimes it is a squared bracket, somethings its round brackets and in this case here its both. I would recommend to unify them and then check if they are all still correct or if because of the different referencing methods, there has been a mix of references.**

We thank the reviewer for this observation, We have updated the referencing style to be consistent with the journal requirements.

**line 119: Are there multiple Bomet Counties?**

There is only one Bomet county. We use the expression Narok and Bomet counties to signify more than one area.

**line 149: Exchange to lumbricoides**

Thank you for the keen observation, this has been corrected.

**line 319: Here, your inclusion criteria is verbal assent or consent. But further up in your ethics, you write about written consent. Which one is true?**

We thank the reviewer for this observation. We have updated the manuscript to read verbal informed consent by the parents or guardians.

**Reviewer #3: The revised manuscript is well written and clearly presented. Figures and tables are appropriate and support the findings effectively. Minor editorial suggestions include ensuring clarity in a few expanded discussion points—particularly around the feasibility and cost implications of expanding PC delivery to preschool-aged children and women of reproductive age. While the authors cite relevant pilot experiences, a more explicit discussion of implementation challenges (e.g., health system integration, community delivery platforms) would further strengthen the operational relevance of the recommendations.**

**These are minor suggestions that do not affect the overall conclusions.**

**Summary and General Comments**

**Use this section to provide overall comments, discuss strengths/weaknesses of the study, novelty, significance, general execution and scholarship. You may also include additional comments for the author, including concerns about dual publication, research ethics, or publication ethics. If requesting major revision, please articulate the new experiments that are needed.**

**Reviewer #1: Methods Ethical Consideration**

**Wouldn't there be a National Ethics Committee in Kenya for ethical approval? Response: Ethical approval was granted by the Amref Health Africa Ethics and Scientific Review Committee, which is accredited by the National Commission for Science, Technology and Innovation (NACOSTI). At the time of the study, this was deemed sufficient to conduct the study**

**Reviewe#1: From now on, you have to go through the National Ethics Committee in addition to the institutional commission.**

We thank the reviewer for their comment. Ethical approval was granted by the Amref Health Africa Ethics and Scientific Review Committee, which is accredited by the National Commission for Science, Technology and Innovation (NACOSTI), the national body. At the time of the study, this was deemed sufficient to conduct the study. Future surveys will seek approval from the National Ethics Committee as per updated national requirements.

**Were children aged 2-4 able to give verbal consent? What was the benefit for participants, particularly those diagnosed as positive?**

**Response: For children aged 2-4 years, verbal assent was provided by the parent/caregiver. While no deworming was provided immediately post-survey, participants received health education and were referred to ongoing local PC programs. This approach is consistent with the survey protocol and ethical approvals.**

**Reviewe#1: This statement comes from you “Written informed consent by parents or guardians and verbal assent by participating children was obtained before enrolment”.**

We thank the reviewer for their comments. For children aged 2-4 years, verbal assent was provided by the parent/caregiver and updated explicitly in the manuscript. While no deworming was provided immediately post-survey, participants received health education and were referred to ongoing local PC programs. This approach is consistent with the survey protocol and ethical approvals.

**Survey setting and population**

**Why did you choose particularly Narok and Bomet for this study? Is there an explanation?**

**What were the criteria used to select the composite locations?**

**Response: We appreciate this observation and query by the reviewer regarding the selection of these counties. These counties were purposively selected due to persistent STH transmission despite multiple rounds of preventive chemotherapy. The composite locations were defined as clusters and sampling as per the ICPSM protocol to ensure representativeness across the three at-risk-groups.**

**Reviewe#1: Please include this information in the manuscript. Also, make the “hotspot” aspect part of the title.**

We thank the reviewer for their comments. As earlier indicated, These counties were purposively selected due to persistent STH transmission despite multiple rounds of preventive chemotherapy. We have updated the criteria in the manuscript as suggested. However, we have not included these areas as hotspots as it doesn’t conform to the WHO definition of hotspots as highlighted in the [WHO M&E](https://iris.who.int/bitstream/handle/10665/379352/9789240099364-eng.pdf?sequence=1&isAllowed=y) [framework for assessing schistosomiasis and soil transmitted helminthiases control](https://iris.who.int/bitstream/handle/10665/379352/9789240099364-eng.pdf?sequence=1&isAllowed=y) [programmes](https://iris.who.int/bitstream/handle/10665/379352/9789240099364-eng.pdf?sequence=1&isAllowed=y)

**The methods section is poorly structured!**

**We appreciate the reviewer’s helpful feedback regarding the structure of the Methods section. In response, we have reorganised and expanded the section to follow a clearer and more logical structure, directly aligned with the ICSPM survey methodology.**

**The revised Methods section now includes the following distinct subheadings:**

1. **Ethics Statement**
2. **Survey Setting and Population**
3. **Survey Design**
4. **Recruitment and Sample Collection**
5. **Laboratory Procedure**
6. **Classification of Infection Intensity**
7. **Data Collection and Management**
8. **Data Analysis**

**Reviewe#1: No major changes in the structure!**

We maintained the methods section as is as this aligns with the [STROBE checklists](https://www.strobe-statement.org/checklists/) for cross sectional analysis and our initial data analysis plan.

**Statistical analysis**

**The statistical analysis section is not rich in information. No statistical tests have been applied! I suggest that you carry out comparative analyses by gender, age group (intra county) and between counties.**

**Reviewe#1: No response was given to this request! This is crucial!!!**

**What did you do with the questionnaire data? Wasn't it possible to highlight risk factors?**

**Response: We thank the reviewer for the comments. As per the ICSPM methodology we did not assess the risk factors in this manuscript. However, the questionnaire data were collected for future risk factor analysis, which was beyond the scope of this baseline prevalence manuscript required for programmatic decision making on PC administration.**

We thank the reviewer for this comment. However as initially indicated, As per ICSPM methodology and our data analysis plan, we did not assess risk factors in this manuscript. This is because the study was powered to classify prevalence thresholds per risk group, not to detect differences between subgroups.

**Reviewe#1: We can't make any programmatic decisions without taking stock of the risk factors... that would be putting the cart before the horse! If the data is there, why not exploit it?**

We thank the reviewer for making this observation. However, programmatic decisions to conduct MDAs, which is the focus of this paper, are based on prevalence thresholds and not the risk factors associated with infection. The risk factors collected are used for other routine decisions which were not the intended focus of this paper.

**Reviewer #2: I think the authors have targeted an important topic, and its important to know the local prevalences of Counties for STH. However, I think the discussion needs a bit more discussion, including what the results mean for the implementation of interventions for the area and how they could be implemented. What is needed to reduce the prevalence in the area and how can this be solved? What does the WHO say and how did other NTD programmes or studies successfully reduced prevalence? What are the implications of your study?**

We thank the reviewer for this comment and we have included other measures required not only to reduce the prevalence but also sustain the gains made through deworming programs.

**Reviewer #3: Major comments**

- **Justification of Sample Size and Design Effect: The authors have now provided sufficient justification for the sample size based on ICSPM (Integrated Community-based Survey for Program Monitoring) and WHO TAS guidelines, explaining that the sample allows for classifying prevalence thresholds with appropriate power. They also clarified the assumed design effect (DEFF = 2.0), consistent with ICSPM methodology. This addresses previous concerns.**
- **Applicability of WHO’s 2% Threshold: The revised manuscript includes a more nuanced discussion about the relevance of the 2% MHI threshold, referencing literature (e.g., Truscott et al., 2021; Werkman et al., 2018) to explain how transmission dynamics may affect interpretation. This addition strengthens the discussion and aligns the study with programmatic and contextual considerations.**
- **Kato-Katz Limitations and Diagnostic Sensitivity: The manuscript now adequately acknowledges that even with mitigation measures (use of local/mobile laboratories), Kato-Katz likely underestimates hookworm prevalence in low-endemicity settings. The proposed references were appropriately included. Future use of more sensitive diagnostics (e.g., PCR) is acknowledged.**
- **Operational Feasibility of Expanding PC to PreSAC and WRA: The authors have expanded the discussion by referencing pilot experiences in Kenya and Malawi.**
- **Consideration of Reinfection and WASH: The revised discussion addresses how poor sanitation and limited access to WASH contribute to persistent transmission and reinfection. This strengthens the public health interpretation and links prevalence findings to broader structural issues.**
- **Interpretation of Prevalence Changes Over Time: While the manuscript notes that the survey was not powered to detect statistically significant changes from earlier surveys, it now clarifies that MHI prevalence remains above elimination thresholds, justifying the need for continued MDA.**
- **Alternative Treatments for Trichuris trichiura: The revised manuscript includes a brief but important mention of ivermectin-albendazole combination therapy, citing recent studies. This addresses concerns about the limited efficacy of albendazole for T. trichiura.**

**Minor Comments**

- **The authors clarified that one-sided upper confidence intervals were used to assess if prevalence is below thresholds, following ICSPM protocol. While two- sided intervals or p-values might enhance comparative interpretation, the current approach aligns with the survey’s programmatic objectives.**
- **The revised ethics section now specifies that participants received health education and referrals to existing programs. This addresses the concern regarding post-survey follow-up in the absence of on-site treatment.**
- **The abstract has been revised to emphasize the main findings and recommendations. The conclusions are now more actionable, suggesting**

**programmatic expansion of PC to underserved groups.**

- **Although a separate figure for co-infections was not included, the data are clearly presented in Table 3. This is acceptable and consistent with the structure of the manuscript.**

We thank the reviewer for the important insights in improving the clarity and potential impact of the findings to the scientific community.

Revised Article with Changes Highlighted

[Click here to access/download](https://www2.cloud.editorialmanager.com/pntd/download.aspx?id=1474876&guid=bc83ffce-2734-4066-a69a-be817f37f6e5&scheme=1)

[**Revised Article with Changes Highlighted** Revised_manuscript with_track_changes_v4_14082025.docx](https://www2.cloud.editorialmanager.com/pntd/download.aspx?id=1474876&guid=bc83ffce-2734-4066-a69a-be817f37f6e5&scheme=1)
