# Supplementary material for: Prevalence and intensity of soil-transmitted helminth infections in Narok and Bomet Counties, Kenya: Evidence from program monitoring
Source: PLoS Negl Trop Dis. 2025 Dec 30;19(12):e0012415. doi: 10.1371/journal.pntd.0012415 (PMC12753061; doi:10.1371/journal.pntd.0012415)
Supplement: Attachment — Submitted filename: Response to Reviewers 03122025.docx [file pntd.0012415.s010.docx]

**Date: 3rd December 2025**

**Dear Reviewers**

We thank you and the reviewers for the thoughtful and constructive feedback provided. Below, we address each of the reviewers' comments, providing clarifications, justifications, and detailing revisions made to the manuscript.

**Methods**

-Are the objectives of the study clearly articulated with a clear testable hypothesis stated?

-Is the study design appropriate to address the stated objectives?

-Is the population clearly described and appropriate for the hypothesis being tested?

-Is the sample size sufficient to ensure adequate power to address the hypothesis being tested?

-Were correct statistical analysis used to support conclusions?

-Are there concerns about ethical or regulatory requirements being met?

Reviewer #1: (No Response)

Reviewer #3: The updated version has satisfactorily addressed the methodological concerns raised in earlier reviews. The study objectives are now clearly articulated within a programmatic surveillance context, and while no formal hypothesis is tested, the aims are appropriately framed for the descriptive design used. The choice of study design has been justified and is suitable to meet the stated objectives. The target populations—preschool-aged children, school-aged children, and women of reproductive age—are clearly described and remain appropriate for assessing prevalence across risk groups. Importantly, the authors have strengthened their justification of sample size, explicitly referencing ICSPM and WHO TAS methodology and clarifying the design effect assumption (DEFF = 2.0), thereby addressing earlier concerns about statistical power. The statistical analyses also follow the ICSPM protocol, with clear rationale for the use of one-sided upper confidence intervals to determine whether prevalence falls below programmatic thresholds, which resolves the previous ambiguity on analytical approach. Ethical procedures have been clarified, with explicit mention of AMREF’s accredited approval, correction of consent/assent wording, and confirmation that participants received health education and referrals, thereby addressing earlier concerns about post-survey follow-up. Overall, the revisions have resolved the methodological issues raised in earlier rounds, and the study now presents a sound and well-justified framework to support its conclusions.

We thank the reviewers for their comments in improving the methodology section.

**Results**

-Does the analysis presented match the analysis plan?

-Are the results clearly and completely presented?

-Are the figures (Tables, Images) of sufficient quality for clarity?

Reviewer #1: (No Response)

Reviewer #3: In the updated version, the results section has been strengthened and addresses the concerns raised in earlier reviews. The analysis presented is consistent with the predefined ICSPM analysis plan and is clearly aligned with the programmatic objectives of classifying prevalence against WHO thresholds. The results are now more clearly and comprehensively presented, with prevalence estimates disaggregated by species, risk group, and infection intensity, and the limitations of the analyses explicitly acknowledged. Tables and figures have been revised for consistency, with corrected numbering, clearer captions, and inclusion of all relevant abbreviations, thereby improving readability. Co-infection data are appropriately summarized in Table 3, which is sufficiently detailed and makes a separate figure unnecessary.
While subgroup comparative analyses were not conducted, the justification based on ICSPM methodology is acceptable; however, a short statement acknowledging that such analyses could be explored in future work would further strengthen the manuscript.
Overall, the results are now complete, clearly presented, and supported by figures and tables of adequate quality.

We thank the reviewer for their comments. We have excluded figure 2 from the revised manuscript. In addition, we have included a statement acknowledging that future sub group analysis could be explored.

**Conclusions**

-Are the conclusions supported by the data presented?

-Are the limitations of analysis clearly described?

-Do the authors discuss how these data can be helpful to advance our understanding of the topic under study?

-Is public health relevance addressed?

Reviewer #1: (No Response)

Reviewer #3: The revised conclusions are well supported by the data presented and directly linked to the findings on prevalence and intensity across risk groups. The limitations are now more clearly described, including diagnostic sensitivity, intra-county heterogeneity, and loss of data from one cluster, addressing earlier concerns about transparency in interpretation. Still, the limitations section could emphasize more explicitly how diagnostic sensitivity and intra-county heterogeneity may affect programmatic interpretation. The discussion has also been expanded to highlight how these findings contribute to understanding persistent transmission despite long-term school-based deworming, and to situate the results within broader regional and global evidence. The authors now explicitly connect the data to programmatic decisions, including the potential expansion of preventive chemotherapy to PSAC and WRA, and acknowledge alternative treatment strategies for T. trichiura.
The discussion on operational feasibility of expanding PC could also benefit from one or two additional sentences on delivery platforms and cost implications. Public health relevance is clearly articulated, emphasizing the need for tailored interventions, integration with WASH, and alignment with WHO 2030 targets.
The conclusions are stronger, more comprehensive, and clearly address both scientific and programmatic implications, resolving the gaps noted in earlier reviews.

We thank the reviewer for their comments to further strengthen the manuscript. We have updated the limitations section to emphasize the intra-county heterogeneity and the diagnostic limitations of kato-katz in low prevalence settings. We also inclided a statement on expansion of PC from school based to community based platforms.

**Editorial and Data Presentation Modifications?**

Use this section for editorial suggestions as well as relatively minor modifications of existing data that would enhance clarity. If the only modifications needed are minor and/or editorial, you may wish to recommend “Minor Revision” or “Accept”.

Reviewer #1: (No Response)

Reviewer #3: The authors have addressed all major concerns raised in previous reviews, including sample size justification, contextualization of the WHO 2% threshold, diagnostic limitations, and expansion of the discussion on reinfection, WASH, and treatment options. Remaining issues are minor:
• While subgroup comparative analyses were not conducted, the justification based on ICSPM methodology is acceptable. However, a short statement acknowledging that such analyses could be explored in future work would strengthen the manuscript.
• The discussion on operational feasibility of expanding PC could benefit from one or two additional sentences on delivery platforms and cost implications, though this is not essential for acceptance.
• The limitations section could emphasize more explicitly how diagnostic sensitivity and intra-county heterogeneity may affect programmatic interpretation.

The remaining issues as stated above have been addressed in the earlier paragraphs and captured appropriately in the manuscript.

**Summary and General Comments**

Use this section to provide overall comments, discuss strengths/weaknesses of the study, novelty, significance, general execution and scholarship. You may also include additional comments for the author, including concerns about dual publication, research ethics, or publication ethics. If requesting major revision, please articulate the new experiments that are needed.

Reviewer #1: (No Response)

Reviewer #3: (No Response)

**Comments made directly on manuscript**

**On table 1:**

We thank the reviewer for the comments made to the manuscript and we have dropped the number and proportion of females providing stool samples for WRA

In addition, we have removed the percentage (%) next to each figure inside the table to reduce overcrowding

**On table 2:**

It was not possible to use directly the Interval Confidence (IC)?

We thank the reviewer for this observation. We decided to use the point prevalence and upper confidence interval to show the possible range of infection prevalence.

Regarding the concern about duplication between the table and figure, we have dropped the figure and retained the table 2.

**On risk factor prospection**

We thank the revewier for this comment and we have included a revised statement in the limitations section highlighting that lack of risk factors may limit some elements of programmatic decision making.
